# Systematic Reconstruction of the Complete Two-Component Sensorial Network in Staphylococcus aureus

**DOI:** 10.1128/mSystems.00511-20

**Published:** 2020-08-18

**Authors:** B. Rapun-Araiz, A. F. Haag, V. De Cesare, C. Gil, P. Dorado-Morales, J. R. Penades, I. Lasa

**Affiliations:** a Laboratory of Microbial Pathogenesis, Navarrabiomed, Complejo Hospitalario de Navarra (CHN)-Universidad Pública de Navarra (UPNA), IDISNA, Pamplona, Spain; b Institute of Infection, Immunity and Inflammation, University of Glasgow, Glasgow, Scotland, United Kingdom; c MRC Protein Phosphorylation and Ubiquitylation Unit, University of Dundee, Dundee, Scotland, United Kingdom; University of Pittsburgh Medical Center

**Keywords:** *Staphylococcus aureus*, regulon, two-component systems

## Abstract

In bacteria, adaptation to changes in the environment is mainly controlled through two-component signal transduction systems (TCSs). Most bacteria contain dozens of TCSs, each of them responsible for sensing a different range of signals and controlling the expression of a repertoire of target genes (regulon). Over the years, identification of the regulon controlled by each individual TCS in different bacteria has been a recurrent question. However, limitations associated with the classical approaches used have left our knowledge far from complete. In this report, using a pioneering approach in which a strain devoid of the complete nonessential TCS network was systematically complemented with the constitutively active form of each response regulator, we have reconstituted the regulon of each TCS of S. aureus in the absence of interference between members of the family. Transcriptome sequencing (RNA-Seq) and proteomics allowed us to determine the size, complexity, and insulation of each regulon and to identify the genes regulated exclusively by one or many TCSs. This gain-of-function strategy provides the first description of the complete TCS regulon in a living cell, which we expect will be useful to understand the pathobiology of this important pathogen.

**IMPORTANCE** Bacteria are able to sense environmental conditions and respond accordingly. Their sensorial system relies on pairs of sensory and regulatory proteins, known as two-component systems (TCSs). The majority of bacteria contain dozens of TCSs, each of them responsible for sensing and responding to a different range of signals. Traditionally, the function of each TCS has been determined by analyzing the changes in gene expression caused by the absence of individual TCSs. Here, we used a bacterial strain deprived of the complete TC sensorial system to introduce, one by one, the active form of every TCS. This gain-of-function strategy allowed us to identify the changes in gene expression conferred by each TCS without interference of other members of the family.

## Abstract (precis)

Bacteria are able to sense environmental conditions and respond accordingly. Their sensorial system relies on pairs of sensory and regulatory proteins, known as two-component systems (TCSs). The majority of bacteria contain dozens of TCSs, each of them responsible for sensing and responding to a different range of signals. Traditionally, the function of each TCS has been determined by analyzing the changes in gene expression caused by the absence of individual TCSs. Here, we used a bacterial strain deprived of the complete TC sensorial system to introduce, one by one, the active form of every TCS. This gain-of-function strategy allowed us to identify the changes in gene expression conferred by each TCS without interference of other members of the family.

## INTRODUCTION

Two-component signal transduction systems (TCSs) are found in organisms of all domains of life (1). In bacteria, they constitute the basic stimulus-response coupling mechanisms to allow bacteria to sense and respond to changes in the environmental conditions (2, 3). A prototypical two-component system contains a histidine kinase (HK), which autophosphorylates on a conserved histidine residue in response to extracellular stimuli. The phosphorylated HK then binds and transfers the phosphoryl group to a conserved aspartate residue on the response regulator (RR) (4–6). The RRs are often DNA-binding transcriptional activators and/or repressors, and the affinity for their DNA targets increases when they are phosphorylated. Thus, the primary consequence of the activation of a TCS is the expression of a specific set of genes controlled by that TCS. In fewer cases, the RR lacks the DNA-binding domain and exerts its regulatory effect by establishing direct interactions with protein or RNA targets and adaptation does not necessarily imply modifications in gene expression (7).

Traditionally, the regulon controlled by an individual TCS has been identified through the comparative analysis of the transcription profiles between the wild type and isogenic mutants in the respective sensor kinase and/or response regulator genes. Despite the tremendous progress made in understanding the functions of individual TCSs, limitations associated with this approach have rendered our current knowledge about the sets of genes controlled by each specific TCS far from complete. For example, in most of the cases, the signal(s) sensed by TCSs remains unknown, and consequently, studies have been conducted in environmental conditions where the TCS might be only partially activated. Thus, changes in gene expression caused by the absence of the TCS might be biased toward those promoters for which the RR shows higher affinity (8). Other limitations include the fact that some genes can be regulated by multiple TCSs. As such, the absence of one of these TCSs may not have an impact on the expression of the regulated genes while the other TCSs are present. An additional and substantial problem that limits our knowledge about the genes controlled by specific TCSs is the fact that very often, studies of different TCSs of the same bacterium have been performed in different laboratories using different bacterial strains that were grown under different environmental conditions. As a consequence of these limitations, many important questions about the TCS network such as the specific and complete regulon controlled by each TCS, the set of genes regulated uniquely by a single TCS, or conversely, the common genes activated by many different TCSs, or the extent by which the regulons of individual TCSs overlap, remain to be determined.

Over the past few years, global questions related with the TCS network have been addressed in the clinically important pathogen Staphylococcus aureus (9–11). This bacterium carries genes that encode 16 TCSs, and only a particular subtype of methicillin-resistant S. aureus (MRSA) strains harbors an additional TCS in the *mec* cassette element (https://www.ncbi.nlm.nih.gov/Complete_Genomes/SignalCensus.html) whose function is linked to the induction of methicillin resistance. Multiple studies have demonstrated the relevance of the different TCSs both in the biology and pathogenesis of S. aureus (12). Thus, TCSs have been involved in virulence (AgrCA and SaeSR) (13–15); antibiotic resistance and cell wall damage (VraRS, GraXRS, and BraSR) (16–21); cell wall metabolism, autolysis, biofilm development, and cell death (WalRK, ArlSR, and LytSR) (22–25); bacterial respiration, fermentation, and nitrate metabolism (SrrBA, AirRS, and NreCAB) (26–28); and nutrient sensing and metabolism (HssRS, KdpDE, and PhoRP) (29–32). In previous work, we deleted all the TCSs present in two different S. aureus strains and demonstrated that among the 16 TCSs, only WalRK is essential for the bacterial growth, while the other TCSs are dispensable and can be deleted simultaneously in the same strain without affecting cell viability (10). In this study, we have complemented the S. aureus strain containing only the essential WalRK TCS (S. aureus ΔXV) with the constitutively active forms of every response regulator (RR*) to characterize the complete TCS regulon. The combination of this genetic reductionist strategy with transcriptome and proteome techniques has allowed us to identify the group of genes whose expression changes in the presence of each TCS. The results revealed that the size of the direct regulons is highly diverse and with few exceptions each TCS regulon always included genes exclusively regulated by the respective TCS. At the same time, the regulons of many TCSs act in concert to control the same subgroup of genes, strongly suggesting that bacterial physiology adapts to different environments using common pathways. These results uncover the commonalities and specificities of each TCS regulon with respect to its partners and provide, for the first time, the complete catalogue of genes controlled by each TCS in S. aureus. We anticipate these results will be of enormous value to understand the biology and virulence of S. aureus and to design strategies to combat this important pathogen.

## RESULTS

### Phosphomimetic response regulators completely activate TCS regulons.

**FIG 1:**
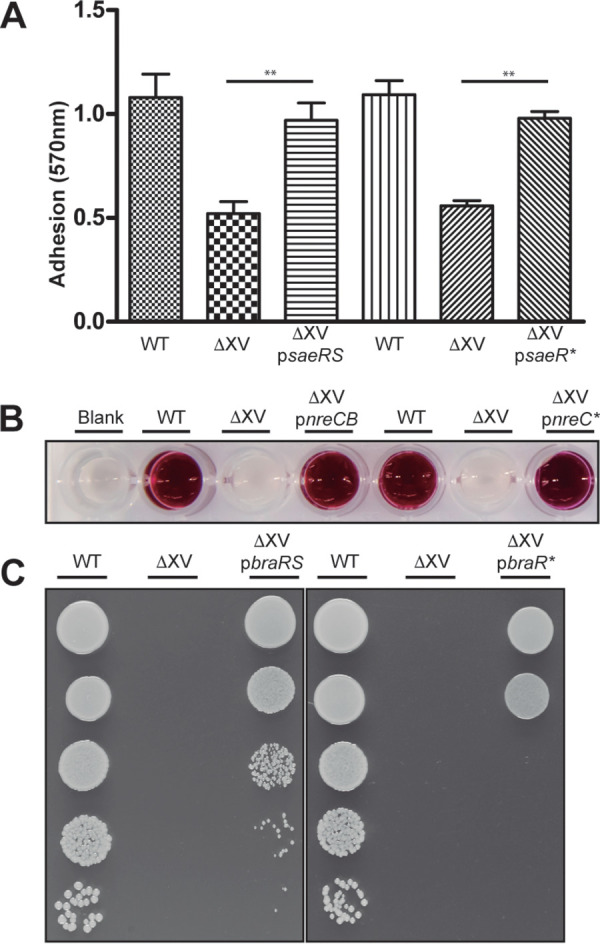
Overproduction of the TCS and RR* and phenotypic restoration. (A) Adhesion to human fibronectin. Wells of ELISA plates were coated with 10 μg ml^−1^ of human fibronectin. Bacteria containing pRMC2 plasmids were induced with 100 ng ml^−1^ of aTC. Data are averages and error bars are standard deviations (SD) for independent triplicate determinations. GraphPad Prism 5.01 was used to analyze and plot the results. Statistical significance was determined by one-way analysis of variance (ANOVA) followed by Bonferroni’s multiple-comparison test and indicated by bars and asterisks as follows: **, *P* < 0.05. WT, wild type. (B) Nitrate reduction capacity. The presence of nitrite was colorimetrically detected. A 10 ng ml^−1^ concentration of aTC was used to induce expression from the pRMC2 plasmids. (C) Bacitracin resistance. Bacterial growth on TSA medium supplemented with bacitracin (5 μg ml^−1^) was assessed by serial dilutions. A 100 ng ml^−1^ concentration of aTC was needed to obtain optimal levels of the BraR* protein.

To identify the set of genes regulated by each TCS in S. aureus and considering that the signals that activate the histidine kinase remain in most cases unknown, we evaluated the efficacy of two different strategies to activate TCS transduction pathways *in vivo*. The first strategy involved expression of the complete histidine kinase and response regulator pair, whereas the second strategy involved the expression of the constitutively active form of the response regulator (RR*) through mutation of the phosphorylation reception residue aspartic acid to a phosphomimetic residue glutamate (33). To confirm whether expression of the different complementation strategies resulted in physiologically relevant gene expression alterations, we next compared the capacity of the native TCS and the RR* to rescue phenotypes associated with three selected TCSs (SaeSR, NreCB, and BraSR) in S. aureus ΔXV. The results showed that both complementation strategies were able to restore the corresponding phenotypes. Thus, SaeR* restored the capacity of the bacteria to adhere to human fibronectin (Fig. 1A), NreC* restored the capacity to reduce nitrate to nitrite (Fig. 1B), and BraR* restored the resistance to bacitracin (Fig. 1C), confirming that both strategies were viable for determining the regulons of the different TCSs.

**FIG 2:**
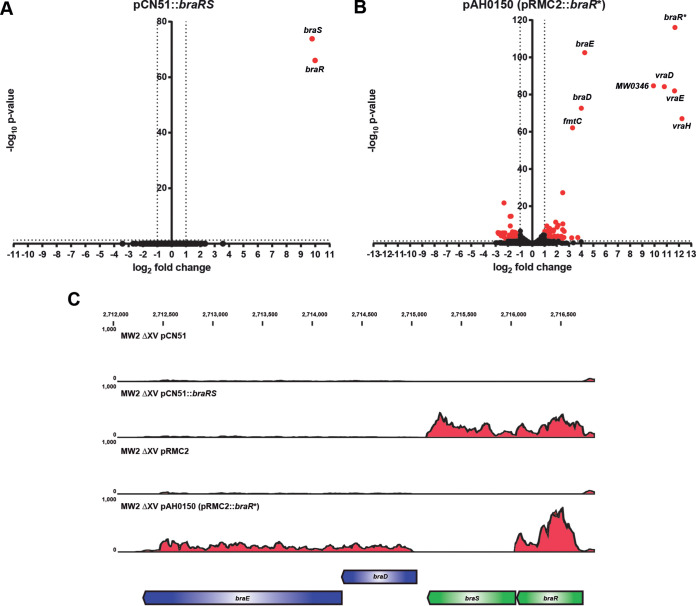
Overproduction of the phosphomimetic RRs leads to higher levels of signal transduction activation. (A) Transcriptomic results of the strain expressing the native version of *braRS* under the control of the P*_cad_* promoter compared with control. (B) Transcriptomic results for the strain complemented with the phosphomimetic form of *braR* under the control of the inducible promoter P*_xyl/tet_* compared to the control. Bacteria were grown at 37°C until exponential phase (OD_600_ of 0.8). Differentially expressed genes were deemed significant for *P* < 0.05 (−log_10_ *P* value > 1.3) and fold change (FC) higher/lower ±2 (log_2_ FC >±1) and are plotted in red. The number of differentially expressed genes is higher in the cells expressing the phosphomimetic RRs. (C) Transcriptomic read maps showing RNA-Seq mapped read distribution of the *braRS* TCS operon and contiguous genes were extracted from CLC Genomics and modified in Adobe Illustrator CS4. A schematic representation of the region of the genome is shown. Open reading frames (ORFs) represented as green arrows correspond to the HK and RR, and ORFs represented as blue arrows correspond to the contiguous genes known to be regulated by BraR. Alignments in the panels from top to bottom correspond to S. aureus MW2 ΔXV carrying either pCN51 (empty control plasmid), pCN51::*braRS* (expressing the complete TCS), pRMC2 (empty control plasmid for expression of phosphomimetic mutants) or pAH0150 (expressing the phosphomimetic version of braR*).

To evaluate the efficiency of both strategies more accurately, we next compared the changes in the transcriptome of cells expressing three selected TCSs (SaeSR, NreCB, and BraSR) or their RR* (SaeR*, NreR*, and BraR*). The differential expression was defined as significant when transcript abundance changed at least twofold with an adjusted *P* value of <0.05 in comparison with the control strain containing the empty plasmid. The results showed that the expression levels and the number of differentially expressed genes was significantly higher in cells complemented with the RR* compared to those complemented with the native TCS. Results in Fig. 2 and Fig. S1 in the supplemental material represented as volcano plots showed that 107, 154, and 264 transcripts increased their relative abundance more than twofold in S. aureus ΔXV complemented with SaeR*, NreR* and BraR*, respectively. Surprisingly, just 4, 12, and 2 transcripts changed their expression more than twofold in S. aureus ΔXV complemented with native SaeSR, NreCB, and BraSR, respectively. Importantly, all the genes whose expression changed when the mutant was complemented with the native TCSs were also identified using the RR* forms.

10.1128/mSystems.00511-20.1 FIG S1 Transcriptomic expression levels of the TCS with the two overexpression strategies. (A and D) Transcriptomic results of the strain expressing the native version of *saeRS* or *nreBC* under the control of the P_cad_ promoter compared to the control. (B and E) Transcriptomic results for the strain complemented with the phosphomimetic form of *saeR* and *nreC* under the control of the inducible promoter P_xyl/tet_ compared to the control. Bacteria were grown at 37°C until exponential phase (OD_600_ of 0.6 to 0.8). Differentially expressed genes were deemed significant for *P* < 0.05 (−log_10_ *P* value > 1.3) and fold change higher/lower ±2 (log_2_ FC > ±1) and were plotted in red. The number of differentially expressed genes is higher in the cells expressing the phosphomimetic RRs (Table 1). (C and F) Transcriptomic read maps showing RNA-Seq mapped reads of the *sae*, *nre*, TCS operons, and contiguous genes were extracted from CLC Genomics and modified in Adobe Illustrator CS4. A schematic representation of the region of the genome is shown. ORFs represented as green arrows correspond to the HK and RR, and the ORFs represented as blue arrows correspond to the contiguous genes known to be regulated by SaeR (C) or NreC (F), respectively. Alignments in the panels from top to bottom correspond to S. aureus MW2 ΔXV carrying either pCN51 (empty control plasmid), pCN51::sae*RS* (C), or pCN51::*nreBC* (F) (expressing the complete TCS), pRMC2 (empty control plasmid for expression of phosphomimetic mutants) or pAH0139 (C) or pAH0149 (F) (expressing the phosphomimetic version of saeR* or nreC*, respectively). Download FIG S1, TIF file, 1.0 MB. Copyright © 2020 Rapun-Araiz et al. 2020 Rapun-Araiz et al. This content is distributed under the terms of the Creative Commons Attribution 4.0 International license.

In accordance with the volcano plots, the normalized log_2_ values representing the number of mapped reads per nucleotide of specific known target genes of each of the three TCSs showed a higher number of reads in genes when the RR* was overproduced in the S. aureus ΔXV compared with the native TCS (see examples in Fig. 2 and Fig. S1). This was even though expression levels of both RR* and the wild-type TCS were comparable (see examples in Fig. 2 and Fig. S1).

Overall, and although both strategies were able to complement the mutant strain phenotypes with the same efficiency, these data indicate that overproduction of the phosphomimetic response regulators is more efficient in activating the corresponding signal transduction pathway than overproduction of the native TCS. Therefore, this strategy was used to unravel the complete regulon of the rest of the TCSs. Another relevant implication of our findings is that genes with fold changes of less than two can still be biologically meaningful.

### Global transcriptional regulon for the two-component sensorial network.

**FIG 3:**
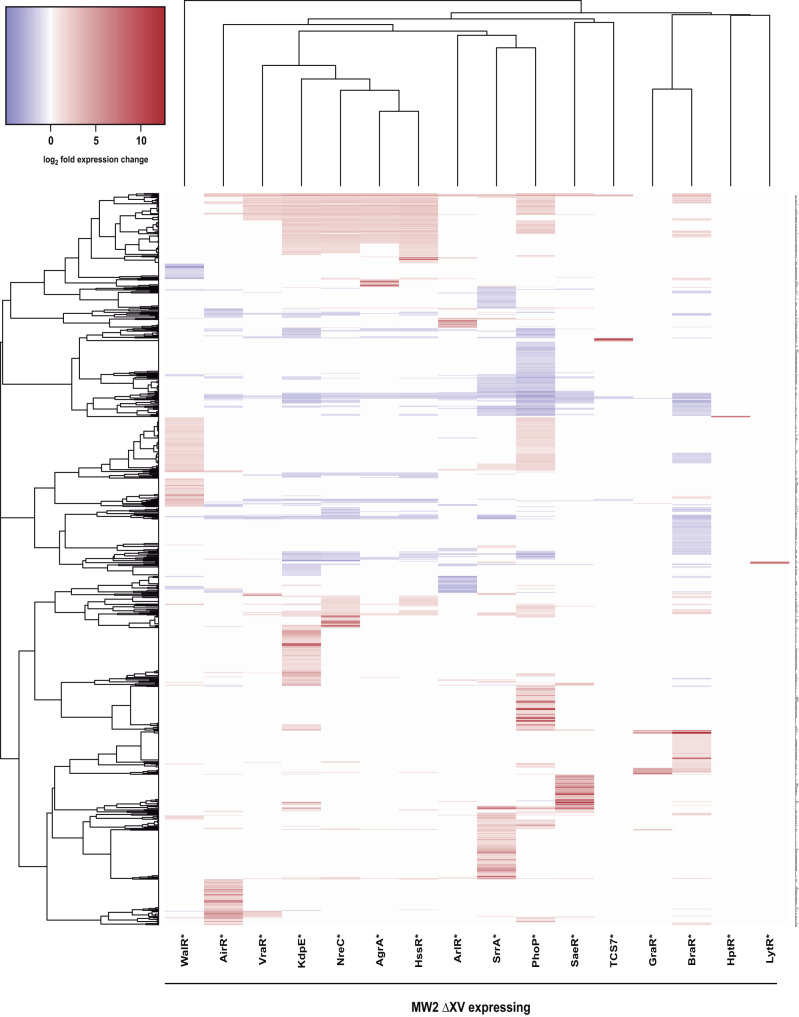
Heatmap of significant gene expression changes. Gene expression changes determined for S. aureus MW2 ΔXV carrying pRMC2 derivatives expressing the indicated phosphomimetic TCS RR* were graphed using heatmap3 in R studio version 1.1.463 and R version 3.5.1. Hierarchical clustering was performed using Euclidian distance and a complete linkage model. The normalized transcriptome data are fully available at our RNA map web browser (http://rnamaps.unavarra.es/).

Given the higher capacity of RR* to activate the signal transduction pathway, we used the collection of 16 S. aureus ΔXV strains complemented with the constitutively active form of each response regulator to identify the complete regulon of the TCS in S. aureus. Overall, we observed 2,443 transcripts in total, affecting 1,075 genes (37% of the S. aureus MW2 genome), changed their expression at least twofold with an adjusted *P* value of <0.05 compared to the control strain containing the empty plasmid (Fig. 3). Of these genes, the expression levels increased for 712 genes and decreased for 306 genes (Table 1).

**TABLE 1:** Number of genes affected by TCS expression^*a*^

Phospho-mimetic RR	No. of genes upregulated by fold change (*P* < 0.05)^*b*^		No. of genes downregulated by fold change (*P* < 0.05)^*b*^		% of genes regulated >|2|^*c*^	% of genes regulated >|4|^*c*^
	>2	>4	<−2	<−4		
WalR	138	31	38	6	6.00	1.26
HptR	2	2	0	0	0.07	0.07
LytR	3	3	0	0	0.10	0.10
GraR	18	15	1	0	0.65	0.51
SaeR	74	53	33	2	3.65	1.87
TCS7	8	7	6	0	0.48	0.24
ArlR	34	20	59	19	3.17	1.33
SrrA	141	63	101	15	8.25	2.66
PhoP	237	64	149	48	13.16	3.82
AirR	96	50	54	3	5.11	1.81
VraR	63	21	24	2	2.97	0.78
AgrA	95	44	40	2	4.60	1.57
KdpE	224	87	103	17	11.15	3.54
HssR	138	52	64	2	6.88	1.84
NreC	154	55	82	8	8.04	2.15
BraR	127	23	137	16	9.00	1.33

a The expression of genes in the sample expressing the defined phosphomimetic response regulator (RR*) were analyzed, and differentially expressed genes were determined relative to the pRMC2 control sample.

b The columns showing the number of genes up- or downregulated by the expression of the indicated RR* using a fold change cutoff of at least >2-fold or >4-fold and a false discovery rate-corrected *P* value of <0.05.

c The columns showing percentages of genes regulated show the proportion of the S. aureus MW2 genome changing its expression at least two- or fourfold.

These results revealed that the TCSs are more prone to modify the bacterial physiology by activating the expression of their targeted genes than repressing their expression. The number of differentially expressed genes by each TCS under the conditions used in this study ranged between 2 genes in HptR* to 386 genes in PhoP* (Fig. S2 and Table 1).

10.1128/mSystems.00511-20.2 FIG S2 Analysis of genes and proteins shared between the whole TCS network. (A, top) Each intersection indicates the genes shared between the TCSs indicated in the respective columns and rows. Shaded in colors are the number of genes regulated by the individual TCSs. The number of genes that are specifically regulated only by the TCS indicated in the column titles are indicated in the bottom panel (see arrows). (B, top) Each intersection indicates the protein shared between the TCS indicated in the respective columns and rows. Shaded in colors are the number of proteins regulated by the individual TCS. The number of proteins that are specifically regulated only by the TCS indicated in the column titles are indicated in the bottom panel (see arrows). The numbers of overlapping genes and proteins were assessed using the free software Venny2.1 (https://bioinfogp.cnb.csic.es/tools/venny/index.html). Download FIG S2, TIF file, 1.4 MB. Copyright © 2020 Rapun-Araiz et al. 2020 Rapun-Araiz et al. This content is distributed under the terms of the Creative Commons Attribution 4.0 International license.

To determine the genes specifically regulated by each TCS, we considered the transcriptomic data of the whole network and excluded those genes affected by another TCS (Fig. S2). The results showed that the regulons of all TCSs contained genes that, with the exception of LytSR and VraRS, are specific for each of them (see Data Set S1 in the supplemental material). Some TCSs affect the expression of few genes (HptSR, LytSR, and TCS7SR), whereas other TCSs regulate the expression of hundreds of genes. TCSs (PhoRP, KdpDE, HssRS, and NreCB) share a significant number of genes with other TCSs, suggesting that various TCSs coincide in the activation or repression of the same physiological pathway(s) to adapt to different environmental conditions (see Table S1 in the supplemental material). In particular, the group of genes whose expression is regulated by several TCSs included purine biosynthesis genes (*pur* operon), as well as iron acquisition (*sirA* and *isdCD*), glycolysis (*gapR*), methionine biosynthesis (*metN1* and *metN2*), and lysine permease (*lysP*) genes, among others (Table S2). Interestingly, the expression of the large majority of genes regulated by multiple TCSs is always affected in the same direction. However, 57 genes showed expression changes in the opposite direction when expressing different RR* (Table S3), suggesting that the expression levels of these genes were finely controlled by the action of several TCSs in response to multiple external and/or internal stimuli. The regulons of HssRS and NreCB presented the maximum overlap sharing 68% of the genes in their respective regulons. Overall, these results uncover the complete transcriptomic regulon under an identical environmental condition and show that each TCS regulates a specific group of genes together with other genes affected by multiple TCSs.

10.1128/mSystems.00511-20.5 TABLE S1 Gene ontology enrichment analysis. Gene ontology enrichment analysis was performed using PantherDB to identify proteins and pathways overrepresented in each of the phosphomimetic response regulator-controlled gene sets. Overrepresentation analysis was performed using Fisher’s exact test, and gene ontology sets showing statistically significant enrichment after false discovery rate correction (*P* < 0.05) are shown at top-level hierarchical clusters. Download Table S1, DOCX file, 0.05 MB. Copyright © 2020 Rapun-Araiz et al. 2020 Rapun-Araiz et al. This content is distributed under the terms of the Creative Commons Attribution 4.0 International license.

10.1128/mSystems.00511-20.6 TABLE S2 Genes regulated uniformly by multiple TCSs. Genes that significantly and uniformly altered expression by more than one phosphomimetic response regulator were identified (*P* < 0.05 and fold change of >2). For each gene, the TCSs positively and negatively affecting gene expression are indicated. Locus tags shown represent different annotation versions of the MW2 genome. Download Table S2, DOCX file, 0.03 MB. Copyright © 2020 Rapun-Araiz et al. 2020 Rapun-Araiz et al. This content is distributed under the terms of the Creative Commons Attribution 4.0 International license.

10.1128/mSystems.00511-20.7 TABLE S3 Genes affected differentially by multiple TCSs. Genes that significantly and differentially altered expression by more than one phosphomimetic response regulator were identified (*P* < 0.05 and fold change of >2). For each gene, the TCSs positively and negatively affecting gene expression are indicated. Locus tags shown represent different annotation versions of the MW2 genome. Download Table S3, DOCX file, 0.03 MB. Copyright © 2020 Rapun-Araiz et al. 2020 Rapun-Araiz et al. This content is distributed under the terms of the Creative Commons Attribution 4.0 International license.

10.1128/mSystems.00511-20.9 DATA SET S1 Genes regulated specifically by each TCS. For each phosphomimetic mutant, the genes specifically regulated only when expressing the defined phosphomimetic RR (*P* < 0.05) were determined and collated into the indicated Excel sheets. Locus tags shown represent different annotation versions of the MW2 genome. Download Data Set S1, XLSX file, 0.06 MB. Copyright © 2020 Rapun-Araiz et al. 2020 Rapun-Araiz et al. This content is distributed under the terms of the Creative Commons Attribution 4.0 International license.

### Global proteome analysis for the two-component network.

**FIG 4:**
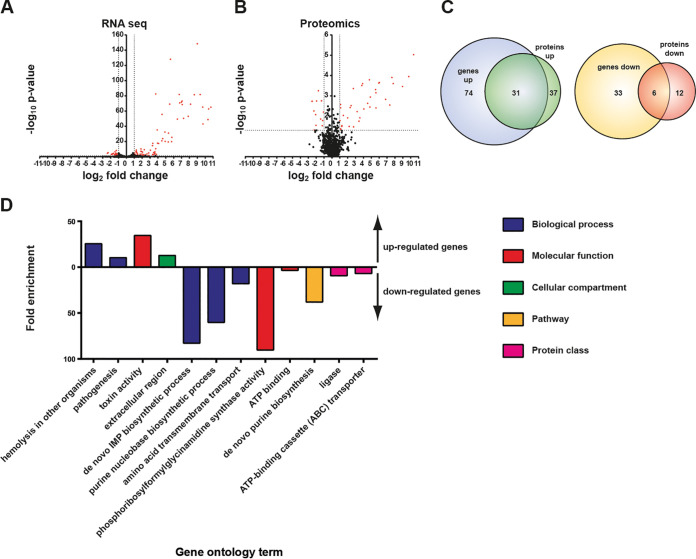
Analysis of the SaeR* TCS regulon. (A and B) Relative gene (A) and protein (B) expression changes between the SaeR*-expressing strain and its pRMC2 control were graphed as volcano plots indicating the log_2_ fold change and the −log_10_ of the FDR-corrected *P* value. (C) The overlap between genes and proteins up- and downregulated in the SaeR*-expressing strain were determined by gene/protein list comparison using visualization from BioVinci version 1.1.5 developed by BioTuring Inc., San Diego, CA, USA. (D) Gene ontology analysis of overrepresented gene ontology terms among upregulated genes (positive bars) and downregulated genes (negative bars). The categories to which the gene ontology terms belong are indicated.

Measuring the abundance of mRNA transcripts in a specific environmental condition provides an incomplete snapshot of the response that the TCSs activate in the cell. Thus, we performed a quantitative profile of protein abundance by mass spectrometry (liquid chromatography-mass spectrometry [LC-MS]) to gain a more complete understanding of the biological processes regulated by each TCS. Differential expression analysis between S. aureus ΔXV and the collection of 16 S. aureus ΔXV strains complemented with each RR* resulted in 267 differentially expressed proteins out of the 1,721 proteins identified in the total lysates. Among them, the expression levels increased for 163 proteins and decreased for 100 proteins, while 4 proteins altered their expression level (positive and negative expression changes) depending on the TCS RR* present (Table S3 and Data Set S2). In most of the TCSs, the number of differentially expressed proteins is lower than the number of genes, except for the regulons of HptR*, LytR*, and GraR*, where the number of differentially expressed proteins is higher (Fig. S2 and Data Set S2). The maximum correlation between the genes and proteins detected in the transcriptomic and proteomic analysis corresponds to 31% of overlap for SaeR* (Fig. 4).

10.1128/mSystems.00511-20.10 DATA SET S2 Number of proteins differentially regulated by the expression of the phosphomimetic TCS RR. Differentially expressed proteins changing their expression level by at least twofold (*P* < 0.05) were identified in the samples expressing the defined TCS phosphomimetic response regulator (RR*). The numbers of proteins positively identified to be either up- or downregulated are shown. Download Data Set S2, XLSX file, 0.10 MB. Copyright © 2020 Rapun-Araiz et al. 2020 Rapun-Araiz et al. This content is distributed under the terms of the Creative Commons Attribution 4.0 International license.

Gene ontology (GO) enrichment analysis confirmed that the majority of genes specifically regulated by the expression of SaeR* were secreted proteins involved in staphylococcal interactions with the host such as toxins targeting immune cells (leukocidins), erythrocytes, and factors of the human immune system. In contrast, genes involved in the *de novo* biosynthesis of purine nucleotides as well as in methionine transport were downregulated in the SaeR*-expressing strain (Fig. 4). Overall, the proteomic subgroup affected by every TCS contained proteins specifically modified by a single TCS, confirming the functional singularity of each TCS (Fig. S2). In agreement with the transcriptomic data, all proteomic expression changes matched the direction of change in the transcriptomic data set (Fig. S3). Together, these data confirm that each TCS modifies the expression of a different panel of genes to adapt bacterial physiology to environmental cues and regulates gene expression at the RNA and protein levels.

10.1128/mSystems.00511-20.3 FIG S3 Overview of the transcriptomic and proteomic expression changes observed when overexpressing each of the phosphomimetic TCSs. Transcriptomic and proteomic expression changes for the strain complemented with the phosphomimetic form of the indicated TCS RR under the control of the inducible promoter P_xyl/tet_ compared with the strain harboring the control plasmid were plotted as volcano plots against the −log_10_ of the FDR-corrected *P* value. Bacteria were grown at 37°C until exponential phase (OD_600_ of 0.6 to 0.8), and differentially expressed genes were deemed significant for *P* < 0.05 (−log_10_ *P* value > 1.3) and fold change higher/lower ±2 (log_2_ FC > ±1) and were plotted in red. To the right of the volcano plots, the overlap between genes and proteins up- and downregulated in the indicated TCS-expressing strain was determined by gene/protein list comparison using visualization from BioVinci version 1.1.5 developed by BioTuring Inc., San Diego, California, USA. Download FIG S3, TIF file, 1.8 MB. Copyright © 2020 Rapun-Araiz et al. 2020 Rapun-Araiz et al. This content is distributed under the terms of the Creative Commons Attribution 4.0 International license.

### Comparative analysis of individual two-component system regulons.

**FIG 5:**
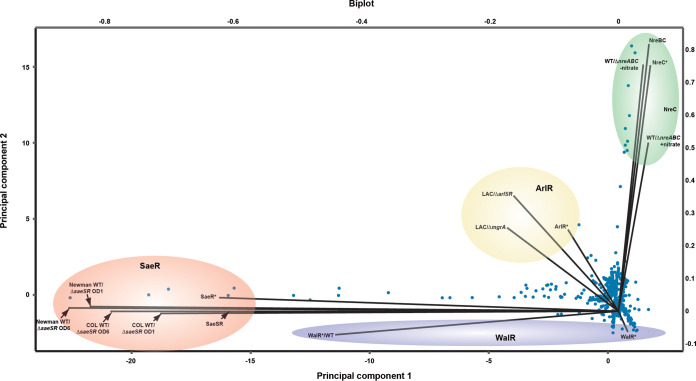
Principal-component analysis comparing phosphomimetic TCS response regulator regulons with published regulons. Differentially expressed genes were extracted from published data sets: regulated genes of the SaeRS TCS comparing the WT strains against clean deletion mutants in *saeRS* in two strain backgrounds (COL and Newman) as well as in two growth phases were extracted from reference 58. Genes affected by the NreBC TCS were extracted from reference 28 comparing the WT against a clean deletion mutant of *nreABC* grown under anaerobic conditions with or without nitrate. Genes affected by the ArlSR TCS are derived from reference 36 comparing the WT against a clean deletion mutant of either *arlRS* or *mgrA*. The published regulons were combined with the data obtained in this study and genes affected by at least one of the TCSs or phosphomimetic RRs indicated were included in the data set analyzed. Principal-component analysis was performed using BioVinci version 1.1.5 developed by BioTuring Inc., San Diego, CA, USA, and eigenvectors of the different data sets analyzed were plotted.

Given that the phosphomimetic form of the RR is constitutively active, we wondered whether complementation with the RR* may exacerbate the response and induce changes in expression of inappropriate genes. Analysis of the regulons of HptSR, LytSR, TCS7SR, GraRS, VraRS, ArlRS, and WalRK comprised 1, 3, 14, 18, 87, 93, and 176 genes, respectively (Table 1). Expression of HptR* resulted in the exclusive upregulation of *uhpT*. *uhpT* is located directly downstream of *hptSR* and transcribed in the opposite direction and encodes the unique glucose 6-phosphate transporter of S. aureus. LytR* affects the expression of only 3 genes, while an early microarray analysis revealed that a LytSR mutation affected the expression of 467 genes (34). Expression of TCS7 RR* resulted in the upregulation of 14 genes, most of them located within the vicinity of the TCS-encoding genes. GraR* activated the expression of 18 genes and downregulated the expression of one gene. Half of these genes were included among the 424 genes differentially expressed in the Δ*graRS* mutant compared with the parental strain (18). VraR* upregulated the expression of 63 genes and downregulated the expression of 24 genes. A previous characterization of the Vra regulon in the presence of vancomycin identified 139 genes (35). Expression of ArlR* activated the expression of 34 genes and downregulated the expression of 59 genes. The ArlRS regulon characterized by comparison of the wild type and its isogenic ArlRS mutant identified 250 genes differentially expressed (36). Forty percent of the genes regulated by ArlRS in our study were also differentially expressed in this study (Fig. 5). Finally, the WalRK system is essential for the bacteria, and the regulon of WalRK was previously characterized using a similar approach of overproducing the phosphomimetic form of WalR* in the wild-type bacteria (37). WalR* activated the expression of 138 genes and downregulated the expression of 38 genes. A substantial number of these genes was confirmed to be activated through the SaeSR TCS in this study indicating that activation of the WalR regulon can result in the activation of the SaeSR and/or other TCS regulons. Of the 176 genes regulated by WalR* in S. aureus ΔXV, 28 were also differentially expressed when WalR* was overexpressed in the wild-type bacteria (37), and importantly, none of the SaeSR-regulated genes were found in our WalR* regulon (Fig. 5).

Overall, these results showed that the sizes of the regulons in our approach are significantly smaller than the regulons previously described, indicating that overexpression of the phosphomimetic form of the RR* does not affect the expression of inappropriate genes. These results also suggested that regulons inferred from the comparison of the wild type and the corresponding TCS mutant include genes that are activated through other TCSs.

### Two-component system-specific fluorescent biosensors.

**FIG 6:**
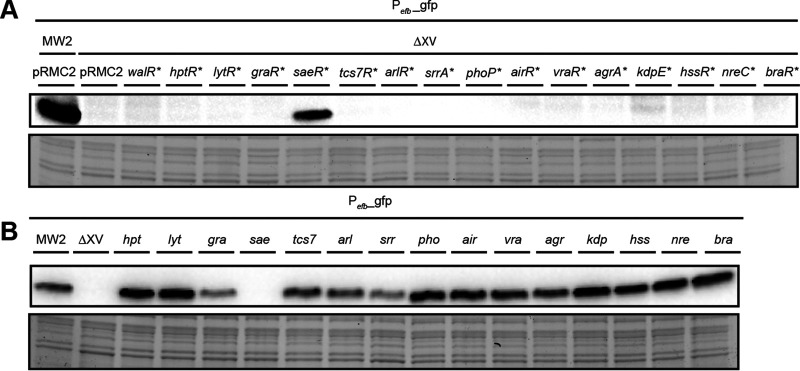
The reporter for *efb* is specifically regulated by its cognate TCS. (A) Representative Western blot showing the GFP levels of P*efb-gfp* plasmid expressed from a collection of S. aureus ΔXV strains containing the indicated RR*. Only when the specific RR* or the genomic copy of the TCS from the wild-type strain is present is the biosensor active. (B) Representative Western blot showing the GFP levels from a collection of single TCS mutants harboring the P*efb-gfp* plasmid. Wild-type S. aureus MW2 and ΔXV were used as controls. GFP expression was observed in all the strains, but the ones lacking the specific TCS responsible for the activation of the genes were studied. Bacteria were grown at 37°C until exponential phase (OD_600_ of 0.8), and when needed, aTC was added at a concentration of 100 ng ml^−1^. A stain-free gel portion is shown as a loading control.

To confirm our transcriptomic analyses and to provide specific tools that can be used to study S. aureus biology, we exploited the transcriptome and proteome analysis shown above. We elucidated the specific subset of genes regulated by each TCS (Table 1 and Data Set S1) and the overlap between the transcriptomic and proteomic results (Data Set S2 and Fig. S3) to generate a collection of reporters specific for each TCS. With the exception of VraR*, we were able to identify a promoter whose expression was significantly higher in the presence of the corresponding RR* for all the TCS (Fig. S4). In the case of *atl* (WalR*), *lrgA* (LytR*), *qox* (SrrA*), and *ctsR* (BraR*), there is residual low-level expression of the reporter in the absence of the inducer (anhydrotetracycline [aTC] [100 ng ml^−1^]). We next evaluated the specificity of each reporter by analyzing the expression of three reporters *efb* (SaeR*), *mgrA* (ArlR*), and *qox* (SrrA*) in the collection of S. aureus ΔXV strains complemented with each RR* (Fig. 6 and Fig. S4). The results confirmed that the activation of the reporter was significant only in the presence of its cognate RR*. Conversely, we used a collection of mutant strains with mutations in each TCS to demonstrate that only in the absence of the specific TCS did the expression levels of the reporter decrease significantly (Fig. 6 and Fig. S4). Together, these data demonstrate the existence of genes whose expression exclusively depends on the presence of a specific TCS, whereas other genes are affected by several TCSs.

10.1128/mSystems.00511-20.4 FIG S4 Generation of a collection of specific biosensors. (A) Representative Western blots showing GFP protein levels expressed from an S. aureus ΔXV strain each containing transcriptional fusions of the target genes selected and the corresponding individual RR*. Different times of exposure were used for the same membrane depending on the biosensor (see bars indicating different exposure times). Bacteria were grown at 37°C until exponential phase (OD_600_ of 0.8), and when needed, aTC was added at a concentration of 100 ng ml^−1^. A stain-free gel portion is shown as a loading control. (B and D) Representative Western blots showing the GFP levels of P*mgrA-gfp* and P*qox-gfp* plasmids expressed from a collection of S. aureus ΔXV strains containing the indicated RR*. (C and E) Representative Western blots showing the GFP levels from a collection of single TCS mutants harboring the, P*mgrA-gfp*, and P*qox-gfp* plasmids. Bacteria were grown at 37°C until exponential phase (OD_600_ of 0.8), and when needed, aTC was added at a concentration of 100 ng ml^−1^. A stain-free gel portion is shown as a loading control. Download FIG S4, TIF file, 2.0 MB. Copyright © 2020 Rapun-Araiz et al. 2020 Rapun-Araiz et al. This content is distributed under the terms of the Creative Commons Attribution 4.0 International license.

## DISCUSSION

Bacteria sense and respond to changes in their environments through two-component signal transduction systems. Free-living bacteria usually contain dozens of TCSs with their number being proportional to the diversity of environments in which organisms live and the complexity in cellular differentiation (38). After 2 decades of intense study, we have a detailed knowledge about the molecular mechanisms underlying TCS signal transduction to the cellular machinery through the phosphotransfer cascade. There is also abundant information about the genes affected by many TCSs, especially those from pathogenic bacteria (12, 39–43). However, a systematic analysis of the functionality of the complete TC sensorial system in the same bacterial cell under signal-independent activation conditions has never been performed. Here, instead of analyzing the effect of the deletion of each TCS, we have analyzed the consequences of introducing one by one the active form of every TCS in a bacterial strain containing only the essential WalRK TCS.

Initially, we compared the efficiency of overexpressing the native TCS or the phosphomimetic RR in activating their corresponding regulons and restore the phenotypes. Our results indicate that the level of activation of the corresponding regulon in both approaches is very different. When the native sensor and response regulator are overproduced, the RR is phosphorylated either by its cognate sensor or by unspecific phosphate donors such as acetyl phosphate or alternative kinases (serine-threonine kinase). These phosphorylated RRs have to withstand the phosphatase activity of the cognate histidine kinase. In contrast, the RR* is insensitive to the phosphatase activity and thus potentially able to reveal target genes that are not the major regulatory targets but that are affected/fine-tuned in the presence of high levels of phosphorylated RR (i.e., when the signaling cascade is activated completely). In addition, the signaling cascade on which activation with the native TCS relies might not be fully active under the growth conditions used to purify the total RNA for transcriptional analysis. These differences might explain why complementation with the native TCS results in very few genes reaching the arbitrary twofold threshold value established to discriminate genes specifically regulated by the TCS from those changes in gene expression due to transcriptional noise. Despite the low number of genes whose expression changes significantly, complementation with native TCS restored the phenotypes as efficiently as complementation with the phosphomimetic RR. These results highlight the well-recognized problem about the adequacy of the fold change cutoff values that are currently used to associate genes to biological phenotypes (44, 45). Identification of the individual TCS regulon is intrinsically dependent on the availability of its cognate signal when assessing transcriptional changes and further emphasizes the need to use culture conditions that activate TCS signaling when determining their target genes. Given that, the signals sensed by staphylococcal TCS are mostly uncharacterized; our approach offers the first systematic characterization of each individual TCS’s regulon that is independent of upstream signaling events. Another uniqueness of our approach is that each RR is analyzed individually in the absence of other members of the family, and therefore, all the changes in gene and protein expression depend specifically on the presence of the constitutively active form of one RR, avoiding sophisticated regulatory networks (46). The most common mechanism for controlling gene expression by RR is through binding to *cis*-regulatory sequences in the promoter of the target gene which affects the recruitment of RNA polymerase for transcription output (8). The finding that a considerable percentage of the genes affected by one RR are also regulated by other RRs confirms the importance of analyzing the regulon of each TCS in the absence of other TCSs to identify unequivocally the genes that are specifically regulated by a TCS from those genes indirectly regulated through another TCS. A limitation of our approach resides in the fact that some RRs regulate biological processes when they are unphosphorylated (47, 48). Understandably, genes regulated by the unphosphorylated form of the RR were not detected in our approach. Future studies may consider identifying the genes regulated by the unphosphorylated form of each RR using the platform developed in this study.

The size of the regulons is highly variable, indicating that some TCSs sense a single ligand, whereas others are more sophisticated and integrate numerous different signals to regulate (a) major lifestyle switch(es), e.g., between virulence, biofilm formation, and cell division. The smallest regulon corresponds to HptSR (49) and affects a single gene, *uhpT*, which is located downstream of the TCS genes and transcribed in the opposite direction. This TCS has remained insulated from other regulatory pathways after acquisition by horizontal transfer (3). A very similar situation occurs with LytSR (50). LytSR regulates mainly the *lrgAB* operon, which is located immediately downstream, strongly suggesting that *lytSR* and *lrgAB* operons form a module that is transmitted horizontally (25). Unlike for HptSR, the regulon of LytSR has not remained insulated, because SrrBA also affected *lrgAB*’s expression. As the number of genes in the regulon increases, the possibility that the same gene belongs to the regulon of more than one TCS also increases. Our results indicated that overlap between regulons of different TCSs ranged from 24% in the regulons with a higher number of genes (PhoRP and KdpDE) to 65% (NreCB and HssRS). An extreme example of overlap is the group of genes regulated by many TCSs. The fact that the expression of some specific genes is affected by many different TCSs strongly suggest that adaptation to different environmental conditions requires the activation of basic housekeeping functions and points toward these genes as attractive targets for combatting S. aureus infections. The requirement of these common pathways cannot be detected through comparisons between the wild type and the corresponding single mutant because in the presence of other TCSs, these pathways will already be active. We are well aware that the existence of promiscuous targets in the regulons of different TCSs implies that different RRs can directly or indirectly recognize and affect the promoter region of these targets.

In conclusion, this study reports the first global analysis of the complete TCS sensorial network using a reductionist approach where we evaluated the contribution of each TCS individually in the same bacterial strain under identical environmental conditions. A noticeable benefit of this strategy compared with all previous studies is that it allows the identification of the specificities and commonalities of the regulon of each TCS without interference from other members of the family. We anticipate that these results will be of enormous value not just for a better understanding of the biology of an important human and animal pathogen but also for the identification of targets that can be used to combat S. aureus infections.

## MATERIALS AND METHODS

### Oligonucleotides, plasmids, bacterial strains, and culture conditions.

Bacterial strains, plasmids, and oligonucleotides (Stabvida and Sigma-Aldrich) used in this study are listed in Table S4 in the supplemental material. Escherichia coli strains XL1-Blue (Stratagene), DC10B, and IM01B (51, 52) were grown in LB medium (Conda-Pronadisa), and Staphylococcus aureus strains were grown in Trypticase soy broth (TSB) supplemented with 0.25% glucose (TSBg) or not supplemented with glucose (TBS) (Conda-Pronadisa and Oxoid). Media, when required for selective growth, were supplemented with the appropriate antibiotics at the following concentrations: erythromycin, 10 μg ml^−1^; ampicillin, 100 μg ml^−1^; chloramphenicol, 20 μg ml^−1^.

10.1128/mSystems.00511-20.8 TABLE S4 Strains, plasmids, and oligonucleotides used in this study. Download Table S4, DOCX file, 0.08 MB. Copyright © 2020 Rapun-Araiz et al. 2020 Rapun-Araiz et al. This content is distributed under the terms of the Creative Commons Attribution 4.0 International license.

### DNA manipulations and bacterial transformation.

General DNA manipulations were performed using standard procedures. Plasmids were purified using the NucleoSpin plasmid miniprep kit (Macherey-Nagel) according to the manufacturer’s protocol. FastDigest and High-Fidelity restriction enzymes (Thermo Scientific and NEB, respectively) and Rapid DNA ligation kit (Thermo Scientific) were used according to the manufacturer’s instructions. Plasmids were transformed into E. coli XL1-Blue, DC10B, or IM01B and S. aureus by electroporation, using previously described protocols (53).

### Generation of phosphomimetic mutants for the individual S. aureus TCS RR.

Phosphomimetic versions of each response regulator were generated by amplifying two overlapping DNA segments by PCR substituting the aspartic acid for the phosphomimetic glutamate codon. The N-terminal fragment also included the *fhuD2* ribosomal binding site substituting the original ribosomal binding site of each TCS RR. Both PCR fragments were fused using standard PCR procedures and cloned into the KpnI and SacI sites of the anhydrotetracycline-inducible expression plasmid pRMC2 (54).

### Generation of full TCS expression constructs and strains.

Full TCS constructs were cloned into pCN51 under the control of a leaky cadmium-inducible promoter and transformed into the S. aureus MW2 ΔXV strain as previously described (10) (Table S4).

### Preparation of RNA for RNA-seq analysis.

S. aureus MW2 ΔXV derivatives carrying either a control plasmid (pRMC2 or pCN51) or a plasmid containing the single phosphomimetic RR or the full TCS, respectively, were grown overnight in TSB at 37°C. Next, 50 ml TSB cultures were inoculated with a starting optical density at 600 nm (OD_600_) of 0.05 and grown to exponential phase (OD_600_ of ∼0.8, full TCS constructs) at 37°C and 250 rpm. Cultures expressing the phosphomimetic RR or containing the empty control plasmid pRMC2 were grown to early exponential phase (OD_600_ of ∼0.3), induced with 100 ng ml^−1^ anhydrotetracycline (aTC), and incubated for 60 min prior to sample collection (final OD_600_ of ∼0.8). Five milliliters of culture was stabilized using RNAprotect bacteria reagent (Qiagen) according to the manufacturer’s instructions. Bacteria were thereafter collected by centrifugation for 10 min at 5,100 × *g* and 4°C. Bacterial pellets were then either directly processed for RNA extraction or stored at −80°C. For RNA extraction, the bacterial pellet was resuspended in 1 ml of TRIzol reagent (Ambion) and lysed in a FastPrep-24 homogenizer (MP Biomedicals) using three cycles of 60 s at 6.5 m s^−1^, followed by 5-min incubation on ice after each cycle. RNA was extracted from the suspension using the PureLink kit (Ambion) applying an on-column DNase digestion step using the RNase-free DNase kit (Qiagen) according to the manufacturer’s instructions. Residual DNA was removed by a second DNase treatment using RQ1 DNase (Promega), followed by RNA purification using the PureLink kit (Ambion) according to the manufacturer’s instructions. RNA quality was assessed by gel electrophoresis.

rRNA was depleted from 8 to 10 μg of extracted RNA using the MicrobeExpress kit (Ambion) according to the manufacturer’s instructions. The concentration of the remaining RNA sample was determined by Nanodrop followed by confirmation of successful rRNA depletion using the Perkin Elmer Labchip GX Touch 24 according to the manufacturer’s instructions. The libraries were prepared using the Illumina TruSeq stranded mRNA kit omitting the poly(A) selection step and sequenced on the Illumina NextSeq 500 using 75-bp single-end reads and ∼10 M reads per sample.

### RNA-seq analysis.

RNA-seq data were analyzed using CLC Genomics Workbench (version 7.5.2), and reads were mapped to the S. aureus MW2 genome (GenBank accession number NC_003923). Data were log_2_ transformed and normalized using quantile normalization on transformed expression values and the quality of replicates assessed by principal-component analysis. After confirming that all replicates clustered together, differential gene expression analysis was performed comparing each of the phosphomimetic RR-expressing samples with the pRMC2-containing control samples. Differential gene expression analysis was performed using the built-in algorithm of CLC Genomics Workbench (version 7.5.2) and the following settings: total count filter cutoff, 5.0; estimate tagwise dispersion, yes; comparison, against reference; reference name, pRMC2; false discovery rate (FDR) corrected, yes. Genes were considered to be significantly regulated when a fold change of more than twofold was observed and the *P* value after FDR correction was below 0.05.

### Preparation of samples for proteomic analysis.

Samples for proteomic analysis were collected at the same time as samples for RNA-seq. For each sample, 1 ml of bacterial culture was added to 250 μl of 5× proteomic lysis buffer (10% sodium dodecyl sulfate [SDS], 50 mM dithiothreitol [DTT], and 5× EDTA-free protease inhibitor cocktail [Roche]), and cells were lysed in a FastPrep-24 homogenizer (MP Biomedicals) using three cycles of 60 s at 6.5 m s^−1^, followed by 15-min incubation at 95°C. The lysed samples were stored at −80°C until further processing. Cysteines were alkylated by addition of 20 mM iodoacetamide and incubation for 20 min at 25°C in the dark, and then the reaction was quenched by addition of 20 mM DTT.

### Proteomic analysis.

One hundred micrograms of protein extract was precipitated adding trichloroacetic acid and sodium deoxycholate to a final concentration of 10% and 0.04%, respectively. The samples were incubated overnight at 4°C and then centrifuged for 10 min at 21,000 × *g* and 4°C to pellet the proteins. The pellet was washed three times with 70% (vol/vol) ethanol, air dried, and then digested by adding 2 μg of trypsin dissolved in 50 mM ammonium bicarbonate solution to the pellet followed by overnight incubation at 37°C. After digestion, the samples were desalted using Oasis columns (Waters) following the manufacturer’s instructions and dried in an Eppendorf Speedvac. Samples were resuspended to a final concentration of 0.2 μg μl^−1^ in 0.1% (vol/vol) formic acid, and 5 μl was used for LC-MS injection. Mass spectrometric analysis was performed on a Linear ion trap-orbitrap hybrid mass spectrometer (Orbitrap-VelosPro; Thermo) coupled to a U3000 rapid separation liquid chromatography (RSLC) high-performance liquid chromatograph (HPLC) (Thermo). Peptides were trapped on a nanoViper Trap column (2 cm by 100 μm C_18_ column; 5 μm, 100 Å (Thermo, catalog no. 164564) then separated on a 50 cm Thermo EasySpray column (ES803) equilibrated with a flow of 300 nl min^−1^ of 3% solvent B. (Solvent A consisted of 2% acetonitrile and 0.1% formic acid. Solvent B consisted of 80% acetonitrile and 0.1% formic acid.) Peptides were eluted with a 112-min gradient from 3% to 99% solvent B.

Data were acquired using a data-dependent “top 20” method, dynamically choosing the most abundant precursor ions from the survey scan, with the “lock mass” option to improve the mass accuracy of precursor ions (lock mass = 445.120024). Full-scan spectra (*m/z* 400 to 1600) were acquired in the orbitrap with resolution *R* = 60,000 at *m/z* 400 (after accumulation to a Fourier transform mass spectrometer [FTMS] full automatic gain control [AGC] target; 1 × 10^6^). The 20 most intense ions, above a specified minimum signal threshold (2,000), based upon a low resolution (*R* = 15,000) preview of the survey scan, were fragmented using a normalized collision energy of 35, an activation time of 10 ms, and recorded in the linear ion trap.

Label-free quantitation (LFQ) was performed using MaxQuant 1.5.8.3. (55) using the following settings: enzyme in use, trypsin/P; allowing for two missed cleavages; carbamidomethyl of cysteines as fixed modification; methionine oxidation, glutamine, and asparagine deamidation specified as variable modifications. Peptide and protein FDR was set at 0.01, the minimal peptide length was 7, and two unique peptides (unique + razor) were required for label-free quantification. Three biological replicates were searched against the MW database containing translated protein sequences of the MW2 reference genome (accession number NC_003923). Data were further processed using Perseus (version 1.5.3.1): a Student’s *t* test (two-tailed, homoscedastic) was performed on the LFQ intensities and proteins with *P* < 0.05 and a fold change of >2-fold were considered significantly altered in abundance.

### Phenotypic characterization.

To study the capacity for phenotypic restoration of SaeSR, NreCB, and BraSR, the S. aureus wild-type strain and ΔXV strain with an empty plasmid (pCN51 or pRMC2) and the ΔXV strain complemented with each TCS or each RR* were used (Table S4). Adhesion to human fibronectin was analyzed for the SaeSR phenotype as follows: overnight cultures were diluted 1:100 in 20 ml of TSBg. Strains containing pRMC2 plasmids were induced at an OD_600_ of 0.3 with aTC at a concentration of 100 ng ml^−1^. Bacteria were grown up to an OD_600_ of 1. Cells were harvested by centrifugation, and pellets were washed and resuspended in a final volume of 20 ml of phosphate-buffered saline (PBS). Enzyme-linked immunosorbent assay (ELISA) plates (96-well plates) (Nunc Maxisorp; ThermoFisher) were coated with 10 μg ml^−1^ of fibronectin (Sigma) and the crystal violet (VRW) measures were performed in an Epoch plate reader (BioTek) after resuspension of the stained wells in 100 μl of a solution of 20% acetone and 80% ethanol. Reduction of nitrate to nitrite was assessed for the NreCB phenotype as previously described (10). In this case, a concentration of 10 ng ml^−1^ of aTC was used to induce the strains expressing the pRMC2 plasmids. To assess the bacitracin resistance of bacteria expressing the BraSR or BraR* TCS, overnight cultures were adjusted to an OD_600_ of 0.4 and serially diluted in TSBg. A volume of 5 μl of diluted cultures was spotted onto Trypticase soy agar (TSA) plates supplemented with bacitracin at a concentration of 5 μg ml^−1^; when needed, aTC was also added at 400 ng ml^−1^ in the overnight cultures and 100 ng ml^−1^ in the agar plates. Plates without antibiotics were also used as growth control of the strains (data not shown). Representative pictures were taken.

### Generation of transcriptional fusions with GFP of the target gene promoters.

To obtain transcriptional fusions, we amplified the promoter regions of the selected target gene for each TCS using the primers specified in Table S4 and cloned these into pCN52 (56). Plasmids were transformed into S. aureus ΔXV strains that already contained the pRMC2 plasmid with the corresponding phosphomimetic version of each RR. Plasmids expressing the transcriptional fusions of the *efb*, *mgrA*, and *qox* genes were also transformed in MW2 wild-type strain and ΔXV strain (with or without pRMC2 plasmid) as controls, together with the collection of single TCS mutants (10). To analyze the expression of the target genes, total protein extracts were recovered at the same conditions as the samples prepared for the transcriptomic and proteomic assays. Protein extracts were analyzed by sodium dodecyl sulfate-polyacrylamide gel electrophoresis (SDS-PAGE) (Bio-Rad) and Western blotting. Green fluorescent protein (GFP) was detected using an anti-GFP antibody (Living Color A.v. monoclonal antibody [JL-8]; Clontech) diluted 1:2,500 in 0.1% PBS−Tween−5% skim milk. Peroxidase-conjugated goat anti-mouse immunoglobulin G (Invitrogen) diluted 1:5,000 in 0.1% PBS−Tween−5% skim milk were used as secondary antibodies. Proteins were detected with the SuperSignal West Pico chemiluminescent substrate (Thermo-Fisher) following the manufacturer’s recommendations.

### Gene ontology analysis.

In order to perform gene ontology analysis on the regulated genes in the S. aureus MW2 ΔXV mutant strains carrying either the control plasmid or a plasmid expressing a phosphomimetic mutant version of the relevant TCS RR, we first annotated and matched the MW2 proteins with their relevant counterparts in the S. aureus strain NCTC8325. Genes were thereafter filtered for their presence on both MW2 and NCTC8325 strains, and regulated gene lists were analyzed using PantherDB (57) to identify proteins and pathways overrepresented in each of the TCS-regulated gene sets. Overrepresentation analysis was performed using Fisher’s exact test and gene ontology sets showing statistically significant enrichment after false discovery rate correction (*P* < 0.05) are shown at top-level hierarchical clusters.

### Data availability.

Transcriptomic data are available on the http://rnamaps.unavarra.es/ server. Raw sequencing data and aligned reads have been submitted to the National Center for Biotechnology Information, BioProject identifier (ID) PRJNA608927.
