# Supplementary figures and images for: Systematic Reconstruction of the Complete Two-Component Sensorial Network in Staphylococcus aureus
Source: mSystems. 2020 Aug 18;5(4):e00511-20. doi: 10.1128/mSystems.00511-20 (PMC7438023; doi:10.1128/mSystems.00511-20)

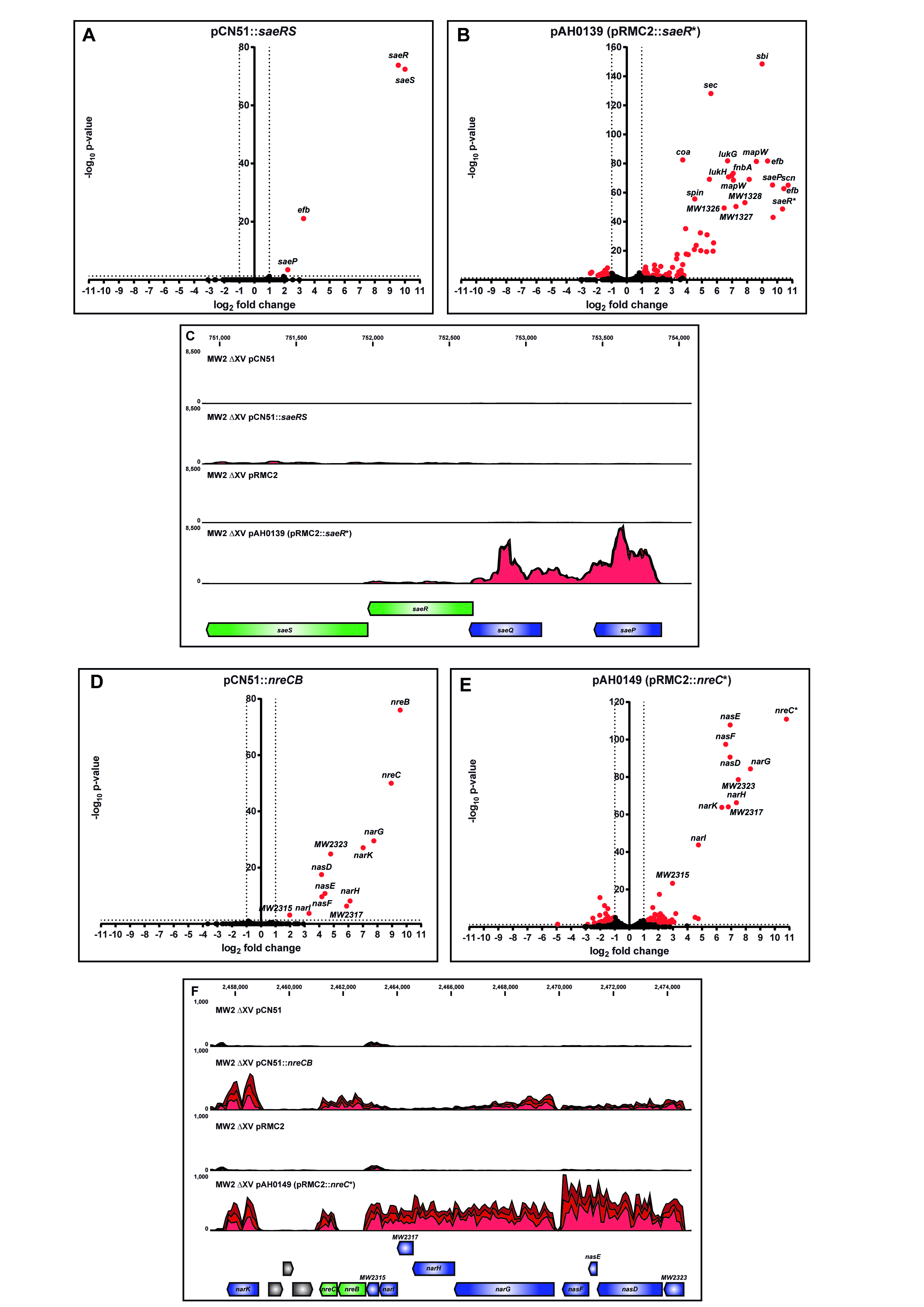

Supplement: FIG S1 [file mSystems.00511-20-sf001.tif]

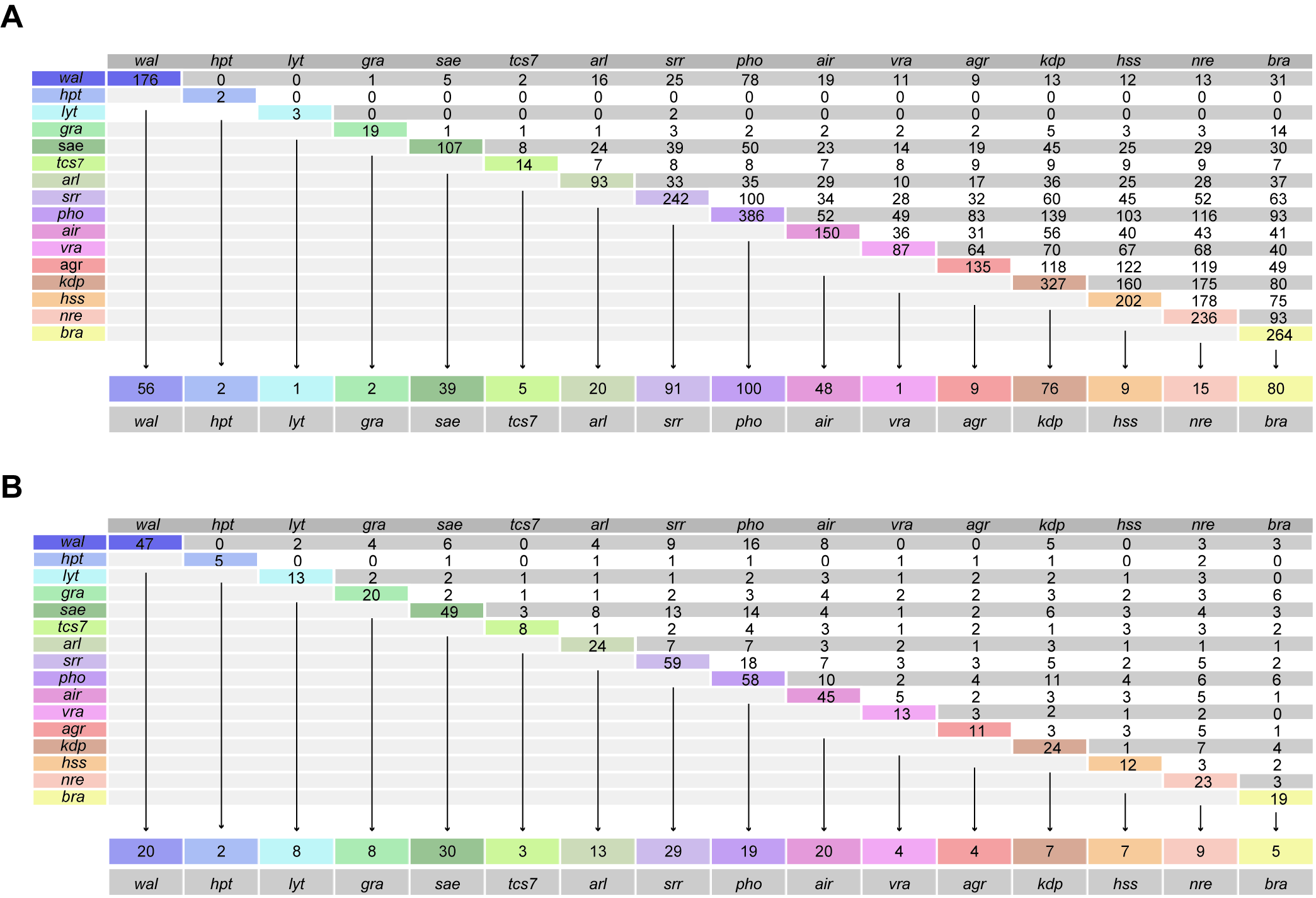

Supplement: FIG S2 [file mSystems.00511-20-sf002.tif]

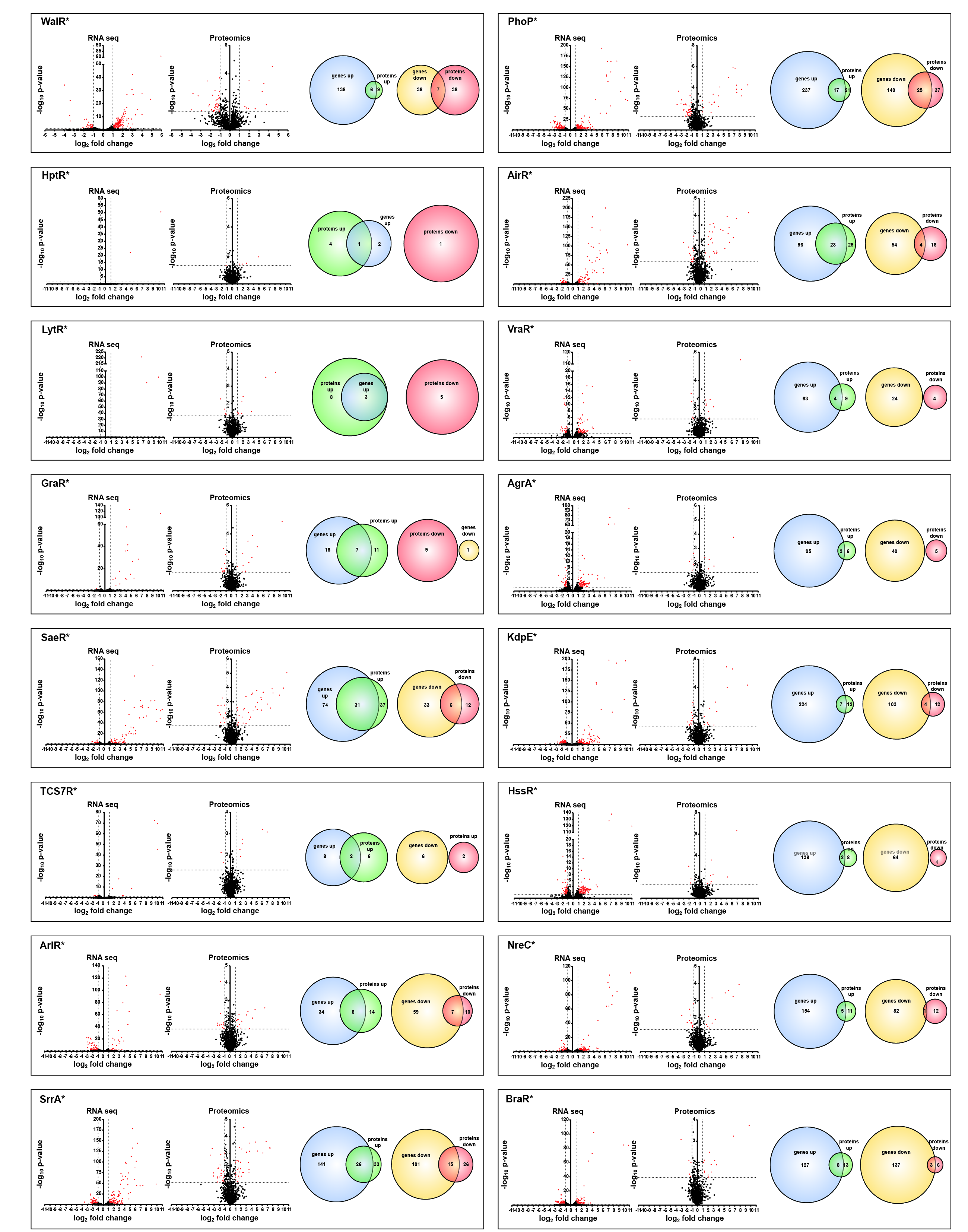

Supplement: FIG S3 [file mSystems.00511-20-sf003.tif]

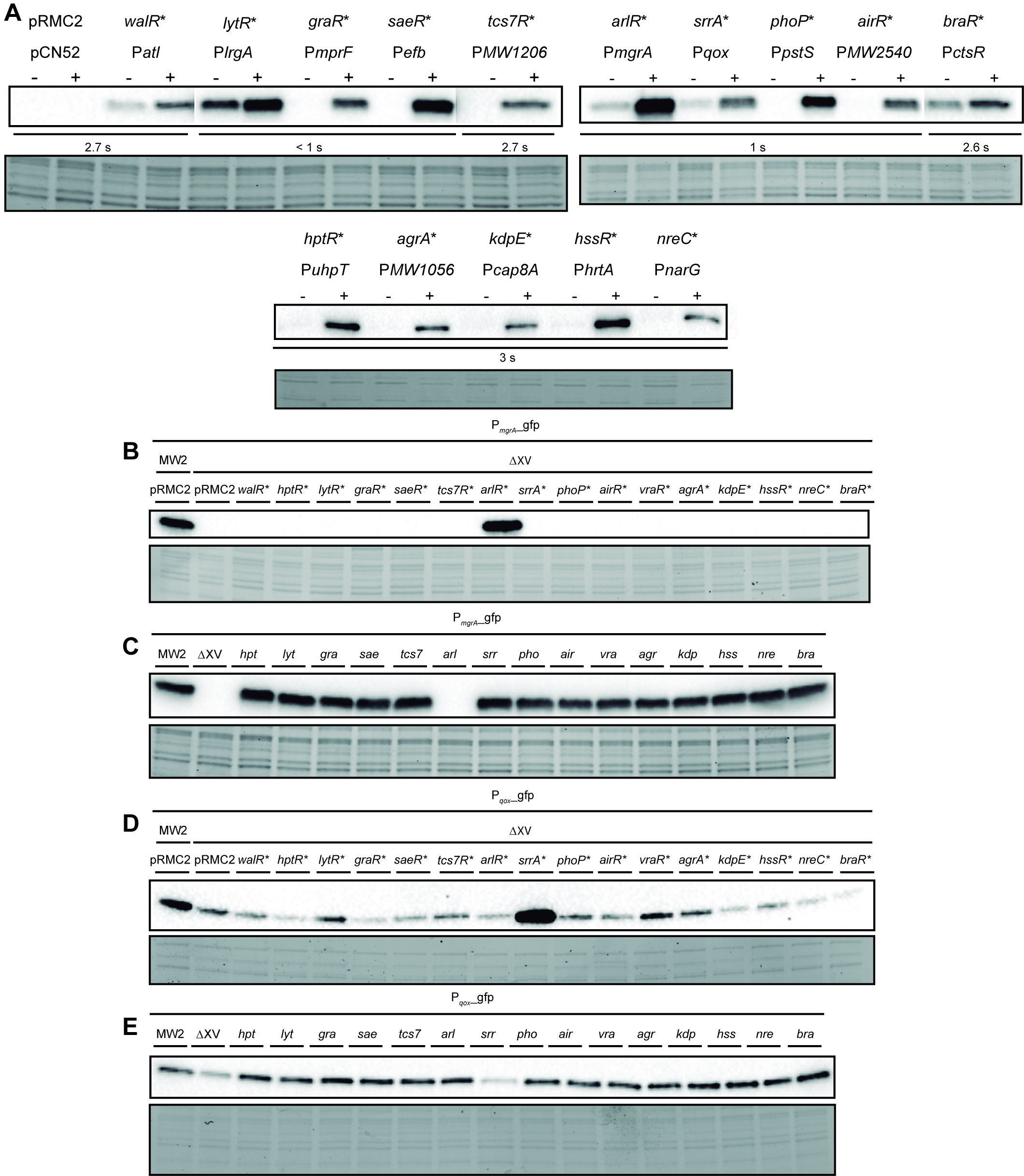

Supplement: FIG S4 [file mSystems.00511-20-sf004.tif]
