# Supplementary material for: Systematic Reconstruction of the Complete Two-Component Sensorial Network in Staphylococcus aureus
Source: mSystems. 2020 Aug 18;5(4):e00511-20. doi: 10.1128/mSystems.00511-20 (PMC7438023; doi:10.1128/mSystems.00511-20)
Supplement: TABLE S1 [file mSystems.00511-20-st001.docx]

**Table S1 Gene ontology enrichment analysis.**

| **Up-regulated genes^a^** | | | |
| --- | --- | --- | --- |
| **WalR*** | **GO biological process complete** | [**Fold Enrichment**](http://pantherdb.org/tools/compareToRefList.jsp?sortOrder=2&sortList=Client%20Text%20Box%20Input&sortField=foldEnrich) | [**FDR**](http://pantherdb.org/tools/compareToRefList.jsp?sortOrder=2&sortList=Client%20Text%20Box%20Input&sortField=fdr) |
| >2-fold | translation | 13.24 | 2.17E-39 |
| >4-fold | defence response to bacterium | 51.59 | 5.04E-02 |
|  | cell wall organization | 12.00 | 1.62E-02 |
|  | **GO molecular function complete** |  |  |
| >2-fold | structural constituent of ribosome | 21.65 | 4.71E-43 |
|  | rRNA binding | 18.56 | 5.67E-26 |
|  | N-acetylmuramoyl-L-alanine amidase activity | 12.74 | 1.69E-02 |
|  | translation factor activity, RNA binding | 10.42 | 2.95E-02 |
|  | tRNA binding | 8.03 | 9.69E-03 |
| >4-fold | N-acetylmuramoyl-L-alanine amidase activity | 57.32 | 6.15E-05 |
|  | **GO cellular component complete** |  |  |
| >2-fold | small ribosomal subunit | 22.93 | 4.61E-06 |
|  | large ribosomal subunit | 22.93 | 7.13E-07 |
|  | **PANTHER Protein Class** |  |  |
| >2-fold | ribosomal protein | 20.96 | 5.60E-26 |
|  | RNA binding protein | 12.45 | 1.39E-25 |
|  | nucleic acid binding | 7.04 | 3.54E-19 |
|  | translation elongation factor | 10.19 | 3.26E-02 |
|  | translation factor | 7.17 | 3.67E-02 |
|  | translation initiation factor | 9.17 | 3.68E-02 |
| **LytR*** | **GO biological process complete** | [**Fold Enrichment**](http://pantherdb.org/tools/compareToRefList.jsp?sortOrder=2&sortList=Client%20Text%20Box%20Input&sortField=foldEnrich) | [**FDR**](http://pantherdb.org/tools/compareToRefList.jsp?sortOrder=2&sortList=Client%20Text%20Box%20Input&sortField=fdr) |
| >2-fold | programmed cell death | > 100 | 1.13E-02 |
| **GraR*** | **GO biological process complete** | [**Fold Enrichment**](http://pantherdb.org/tools/compareToRefList.jsp?sortOrder=2&sortList=Client%20Text%20Box%20Input&sortField=foldEnrich) | [**FDR**](http://pantherdb.org/tools/compareToRefList.jsp?sortOrder=2&sortList=Client%20Text%20Box%20Input&sortField=fdr) |
| >2-fold | lipoteichoic acid biosynthetic process | > 100 | 1.99E-04 |
| **SaeR*** | **GO biological process complete** | [**Fold Enrichment**](http://pantherdb.org/tools/compareToRefList.jsp?sortOrder=2&sortList=Client%20Text%20Box%20Input&sortField=foldEnrich) | [**FDR**](http://pantherdb.org/tools/compareToRefList.jsp?sortOrder=2&sortList=Client%20Text%20Box%20Input&sortField=fdr) |
| >2-fold | hemolysis in other organism | 25.4 | 7.06E-03 |
|  | pathogenesis | 10.26 | 2.62E-12 |
|  | **GO molecular function complete** |  |  |
| >4-fold | toxin activity | 34.39 | 2.22E-02 |
|  | **GO cellular component complete** |  |  |
| >2-fold | extracellular region | 12.7 | 3.34E-1 |
| **ArlR*** | **GO cellular component complete** | **Fold Enrichment** | **FDR** |
| >2-fold | integral component of membrane | 2.44 | 1.80E-01 |
| **SrrA*** | **GO biological process complete** | **Fold Enrichment** | **FDR** |
| >2-fold | ATP synthesis coupled proton transport | 22.05 | 3.83E-05 |
|  | protoporphyrinogen IX biosynthetic process | 13.78 | 3.89E-03 |
|  | arginine biosynthetic process | 10.02 | 1.01E-02 |
|  | anion transport | 4.1 | 2.87E-02 |
|  | oxidation-reduction process | 2.93 | 1.45E-04 |
| >4-fold | electron transport chain | 14.33 | 4.23E-02 |
|  | **GO molecular function complete** |  |  |
| >2-fold | proton-transporting ATP synthase activity, rotational mechanism | 22.05 | 3.78E-05 |
|  | oxidoreductase activity | 3.03 | 1.41E-04 |
| >4-fold | cytochrome-c oxidase activity | 51.59 | 4.86E-02 |
|  | oxidoreductase activity, acting on diphenols and related substances as donors, oxygen as acceptor | 51.59 | 3.64E-02 |

| **SrrA*** | **GO cellular component complete** | **Fold Enrichment** | **FDR** |
| --- | --- | --- | --- |
| >2-fold | proton-transporting ATP synthase complex, coupling factor F(o) | 22.05 | 1.32E-02 |
|  | proton-transporting ATP synthase complex, catalytic core F(1) | 22.05 | 4.44E-04 |
|  | plasma membrane | 1.84 | 3.25E-02 |
|  | integral component of membrane | 1.51 | 1.87E-02 |
| >4-fold | respirasome | 51.59 | 6.14E-02 |
|  | integral component of membrane | 1.8 | 5.35E-02 |
| **SrrA*** | **PANTHER Pathways** | **Fold Enrichment** | **FDR** |
| >2-fold | ATP synthesis | 22.05 | 3.19E-02 |
|  | Heme biosynthesis | 11.03 | 4.74E-03 |
| **PhoP*** | **GO biological process complete** | **Fold Enrichment** | **FDR** |
| >2-fold | phosphate ion transport | 11.36 | 7.34E-03 |
|  | translation | 6.56 | 9.41E-19 |
| >4-fold | phosphate ion transport | 41.97 | 6.01E-05 |
| >100-fold | organic phosphonate transport | >100 | 4.65E-04 |
|  | phosphate ion transmembrane transport | >100 | 2.25E-02 |
|  | **GO molecular function complete** |  |  |
| >2-fold | structural constituent of ribosome | 10.55 | 1.34E-22 |
|  | rRNA binding | 10.41 | 4.79E-17 |
| >100-fold | organic phosphonate transmembrane transporter activity | > 100 | 2.06E-03 |
|  | inorganic phosphate transmembrane transporter activity | > 100 | 1.80E-03 |
|  | **GO cellular component complete** |  |  |
| >2-fold | small ribosomal subunit | 13.25 | 1.55E-04 |
|  | large ribosomal subunit | 13.25 | 4.01E-05 |
| >100-fold | integral component of plasma membrane | 12.38 | 5.35E-02 |
|  | **PANTHER Protein Class** |  |  |
| >2-fold | ribosomal protein | 10.6 | 6.92E-15 |
| **KdpE*** | **GO biological process complete** | **Fold Enrichment** | **FDR** |
| >100-fold | potassium ion transport | > 100 | 4.66E-04 |
|  | **GO molecular function complete** |  |  |
| >10-fold | potassium transmembrane transporter activity, phosphorylative mechanism | 83.34 | 2.51E-02 |
| >100-fold | potassium ion transport | > 100 | 4.66E-04 |
|  | **GO cellular component complete** |  |  |
| >100-fold | integral component of plasma membrane | 34.67 | 6.67E-03 |
|  | **PANTHER Protein Class** |  |  |
| >2-fold | oxidoreductase | 2.74 | 7.39E-04 |
| >10-fold | epimerase/racemase | 14.82 | 2.02E-02 |
| **HssR*** | **PANTHER Protein Class** | **Fold Enrichment** | **FDR** |
| >2-fold | dehydrogenase | 3.4 | 2.36E-02 |
| **NreC*** | **GO biological process complete** | **Fold Enrichment** | **FDR** |
| >2-fold | secondary alcohol metabolic process | 14.43 | 4.15E-02 |
|  | oxidation-reduction process | 2.68 | 1.28E-02 |
| >4-fold | nitrate metabolic process | 57.78 | 7.77E-03 |
|  | oxidation-reduction process | 3.83 | 1.45E-02 |
| **NreC*** | **GO biological process complete** | **Fold Enrichment** | **FDR** |
| >20-fold | nitrate assimilation | > 100 | 3.69E-02 |
|  | oxidation-reduction process | 8.6 | 2.28E-03 |
|  | **GO molecular function complete** |  |  |
| >2-fold | oxidoreductase activity | 2.89 | 2.74E-03 |
| >4-fold | nitrate reductase activity | 57.78 | 3.47E-02 |
| >20-fold | nitrite reductase [NAD(P)H] activity | > 100 | 2.18E-02 |
|  | nitrate reductase activity | > 100 | 6.26E-04 |
|  | **GO molecular function complete** |  |  |
| >20-fold | iron-sulfur cluster binding | 22.57 | 4.78E-02 |
|  | **GO cellular component complete** |  |  |
| >4-fold | nitrate reductase complex | 57.78 | 1.06E-02 |
| >20-fold | nitrate reductase complex | > 100 | 1.27E-04 |
|  | **PANTHER Protein Class** |  |  |
| >2-fold | reductase | 3.96 | 1.58E-02 |
|  | dehydrogenase | 3.8 | 6.89E-04 |
| >4-fold | dehydrogenase | 4.76 | 4.09E-02 |
|  | dehydrogenase | 11.33 | 1.19E-02 |
| **BraR*** | **GO cellular component complete** | **Fold Enrichment** | **FDR** |
| >2-fold | integral component of membrane | 1.87 | 2.68E-04 |
|  |  |  |  |
| **Down-regulated genes^b^** | | | |
| **WalR*** | **GO molecular function complete** | [**Fold Enrichment**](http://pantherdb.org/tools/compareToRefList.jsp?sortOrder=2&sortList=Client%20Text%20Box%20Input&sortField=foldEnrich) | [**FDR**](http://pantherdb.org/tools/compareToRefList.jsp?sortOrder=2&sortList=Client%20Text%20Box%20Input&sortField=fdr) |
| >2-fold | nickel cation binding | 47.52 | 3.91E-04 |
| **SaeR*** | **GO biological process complete** | [**Fold Enrichment**](http://pantherdb.org/tools/compareToRefList.jsp?sortOrder=2&sortList=Client%20Text%20Box%20Input&sortField=foldEnrich) | [**FDR**](http://pantherdb.org/tools/compareToRefList.jsp?sortOrder=2&sortList=Client%20Text%20Box%20Input&sortField=fdr) |
| >2-fold | de novo IMP biosynthetic process | 82.76 | 7.80E-14 |
|  | purine nucleobase biosynthetic process | 60.19 | 3.73E-02 |
|  | amino acid transmembrane transport | 18.06 | 3.27E-02 |
|  | **GO molecular function complete** |  |  |
| >2-fold | phosphoribosylformylglycinamidine synthase activity | 90.28 | 9.07E-03 |
|  | ATP binding | 3.62 | 3.64E-02 |
|  | **PANTHER Pathways** |  |  |
| >2-fold | *de novo* purine biosynthesis | 38.01 | 1.03E-08 |
|  | **PANTHER Protein Class** |  |  |
| >2-fold | ligase | 9.5 | 2.43E-03 |
|  | ATP-binding cassette (ABC) transporter | 6.94 | 2.19E-02 |
| **TCS7R*** | **GO biological process complete** | **Fold Enrichment** | **FDR** |
| >2-fold | de novo IMP biosynthetic process | >100 | 3.51E-03 |
| **ArlR*** | **GO biological process complete** | **Fold Enrichment** | **FDR** |
| >2-fold | urea catabolic process | 50.68 | 1.48E-02 |
|  | de novo IMP biosynthetic process | 25.34 | 9.80E-04 |
|  | peptide transport | 10.14 | 1.67E-02 |
| >4-fold | urea catabolic process | >100 | 1.73E-03 |
|  | **GO molecular function complete** |  |  |
| >2-fold | urease activity | 50.68 | 5.12E-02 |
|  | phosphoribosylformylglycinamidine synthase activity | 50.68 | 3.84E-02 |
|  | nickel cation binding | 38.01 | 1.25E-04 |
| >4-fold | urease activity | >100 | 1.92E-03 |
|  | nickel cation binding | 84.97 | 4.49E-04 |
| **SrrA*** | **GO biological process complete** | **Fold Enrichment** | **FDR** |
| >2-fold | iron import into cell | 28.6 | 1.88E-02 |
|  | diaminopimelate biosynthetic process | 28.6 | 3.04E-03 |
|  | threonine biosynthetic process | 23.84 | 9.48E-04 |
|  | methionine biosynthetic process | 23.84 | 9.13E-04 |
|  | cofactor catabolic process | 22.88 | 4.64E-03 |
|  | lysine biosynthetic process via diaminopimelate | 20.43 | 1.21E-03 |
|  | glycine decarboxylation via glycine cleavage system | 17.16 | 4.10E-02 |
|  | de novo IMP biosynthetic process | 16.69 | 1.29E-04 |
| **SrrA*** | **GO biological process complete** | **Fold Enrichment** | **FDR** |
| >2-fold | cellular aldehyde metabolic process | 14.3 | 5.20E-02 |
|  | carboxylic acid transmembrane transport | 7.53 | 2.53E-02 |
|  | glutamine family amino acid metabolic process | 5.27 | 1.84E-02 |
|  | oxidation-reduction process | 3.5 | 1.75E-05 |
|  | nitrogen compound transport | 3.48 | 3.86E-02 |
| >4-fold | nitrogen compound transport | 14.64 | 3.65E-03 |
|  | organic substance transport | 9.85 | 6.47E-03 |
|  | **GO molecular function complete** |  |  |
| >2-fold | oxidoreductase activity, acting on the CH-NH2 group of donors | 15.89 | 1.21E-02 |
|  | heme binding | 12.26 | 9.83E-03 |
|  | **GO cellular component complete** |  |  |
| >2-fold | plasma membrane | 2.19 | 3.75E-02 |
| >4-fold | plasma membrane | 4.39 | 3.12E-02 |
| ***** | **PANTHER Protein Class** |  |  |
| >2-fold | Threonine biosynthesis | 23.84 | 1.03E-03 |
|  | transporter | 4.01 | 2.55E-06 |
| >4-fold | ATP-binding cassette (ABC) transporter | 13.89 | 1.31E-03 |

| **PhoP*** | **GO biological process complete** | **Fold Enrichment** | **FDR** |
| --- | --- | --- | --- |
| >2-fold | diaminopimelate biosynthetic process | 20.49 | 7.38E-03 |
|  | *de novo* IMP biosynthetic process | 18.78 | 1.98E-06 |
| >4-fold | *de novo* IMP biosynthetic process | 52.34 | 9.38E-11 |
|  | sulfur amino acid metabolic process | 16.75 | 7.14E-03 |
|  | alpha-amino acid biosynthetic process | 5.46 | 3.24E-02 |
| **PhoP*** | **GO biological process complete** | **Fold Enrichment** | **FDR** |
| >4-fold | transmembrane transport | 2.91 | 3.65E-02 |
|  | **GO molecular function complete** |  |  |
| >2-fold | catalytic activity | 1.47 | 4.73E-02 |
| >4-fold | phosphoribosylformylglycinamidine synthase activity | 62.8 | 4.06E-02 |
|  | **PANTHER Pathways** |  |  |
| >2-fold | Threonine biosynthesis | 13.66 | 1.67E-02 |
|  | *de novo* purine biosynthesis | 8.63 | 6.23E-04 |
| >4-fold | *de novo* purine biosynthesis | 23.14 | 5.03E-06 |
|  | **PANTHER Protein Class** |  |  |
| >2-fold | ATP-binding cassette (ABC) transporter | 4.1 | 1.57E-03 |
|  | hydrolase | 2.13 | 4.95E-02 |
| >4-fold | ATP-binding cassette (ABC) transporter | 5.8 | 2.47E-02 |
|  | Lyase | 5.46 | 2.49E-02 |
| **AirR*** | **GO biological process complete** | **Fold Enrichment** | **FDR** |
| >2-fold | de novo IMP biosynthetic process | 29.48 | 3.95E-04 |
|  | nitrogen compound transport | 5.58 | 2.91E-02 |
|  | **GO cellular component complete** |  |  |
| >2-fold | cell wall | 14.74 | 1.61E-02 |
|  | **PANTHER Protein Class** |  |  |
| >2-fold | transporter | 3.95 | 1.03E-02 |
| **AgrA*** | **GO biological process complete** | **Fold Enrichment** | **FDR** |
| >2-fold | *de novo* IMP biosynthetic process | 30.09 | 6.34E-04 |
|  | **GO molecular function complete** |  |  |
| >2-fold | phosphoribosylformylglycinamidine synthase activity | 72.23 | 1.78E-02 |
|  | hydrolase activity, acting on carbon-nitrogen (but not peptide) bonds, in linear amides | 16.05 | 4.33E-03 |
| **KdpE*** | **GO biological process complete** | **Fold Enrichment** | **FDR** |
| >2-fold | de novo IMP biosynthetic process | 30.09 | 1.89E-09 |
|  | purine nucleobase metabolic process | 22.57 | 3.59E-02 |
|  | riboflavin biosynthetic process | 22.57 | 3.30E-02 |
|  | amino acid transmembrane transport | 10.03 | 1.47E-02 |
|  | **GO molecular function complete** |  |  |
| >2-fold | hydrolase activity, acting on carbon-nitrogen (but not peptide) bonds, in linear amides | 8.92 | 5.72E-03 |
|  | **PANTHER Pathways** |  |  |
| >2-fold | flavin biosynthesis | 24.07 | 3.69E-03 |
|  | *de novo* purine biosynthesis | 14.25 | 6.95E-06 |
|  | **PANTHER Protein Class** |  |  |
| >2-fold | transporter | 3.12 | 4.54E-03 |
| **HssR*** | **GO biological process complete** | **Fold Enrichment** | **FDR** |
| >2-fold | de novo IMP biosynthetic process | 19.42 | 5.58E-03 |
|  | **PANTHER Protein Class** |  |  |
| >2-fold | transporter | 3.41 | 2.28E-02 |
| **NreC*** | **GO biological process complete** | **Fold Enrichment** | **FDR** |
| >2-fold | *de novo* IMP biosynthetic process | 31.27 | 6.08E-08 |
|  | iron ion transport | 17.05 | 2.27E-03 |
|  | **PANTHER Pathways** |  |  |
| >2-fold | *de novo* purine biosynthesis | 13.82 | 1.76E-04 |

| **BraR*** | **GO biological process complete** | **Fold Enrichment** | **FDR** |
| --- | --- | --- | --- |
| >2-fold | urea catabolic process | 21.72 | 2.97E-02 |
|  | de novo IMP biosynthetic process | 19.91 | 7.71E-08 |
|  | riboflavin biosynthetic process | 16.29 | 4.33E-02 |
|  | ATP synthesis coupled proton transport | 10.86 | 2.72E-02 |
|  | aspartate family amino acid biosynthetic process | 7.76 | 2.24E-02 |
|  | translation | 3.81 | 1.73E-04 |
|  | nitrogen compound transport | 3.52 | 6.84E-03 |
|  | organic substance transport | 2.57 | 4.43E-02 |
| >4-fold | *de novo* IMP biosynthetic process | > 100 | 2.28E-14 |
|  | heme transport | > 100 | 6.74E-03 |
|  | glutamine metabolic process | 37.52 | 4.94E-02 |
| **BraR*** | **GO molecular function complete** | **Fold Enrichment** | **FDR** |
| >2-fold | rRNA binding | 7.24 | 1.20E-05 |
|  | structural constituent of ribosome | 6.03 | 2.22E-05 |
|  | ATP binding | 2.3 | 2.29E-03 |
|  | metal ion binding | 2.03 | 3.79E-02 |
|  | hydrolase activity | 1.86 | 3.68E-02 |
| >4-fold | phosphoribosylformylglycinamidine synthase activity | > 100 | 6.88E-04 |
|  | **GO cellular component complete** |  |  |
| >2-fold | proton-transporting ATP synthase complex, catalytic core F(1) | 13.03 | 2.93E-02 |
|  | ribosomal subunit | 7.24 | 1.37E-02 |
|  | **PANTHER Pathways** |  |  |
| >2-fold | Flavin biosynthesis | 17.38 | 8.37E-03 |
|  | *de novo* purine biosynthesis | 10.29 | 1.06E-04 |
| >4-fold | *de novo* purine biosynthesis | 65.17 | 4.06E-08 |
|  | **PANTHER Protein Class** |  |  |
| >2-fold | ribosomal protein | 8.07 | 1.76E-06 |
|  | transporter | 2.52 | 1.07E-02 |
| >4-fold | ligase | 18.1 | 6.85E-04 |

^a^No overrepresented GO among up-regulated genes were found in HptR*, TCS7R*, AirR*, VraR* and AgrA*

^b^No overrepresented GO among down-regulated genes were found in HptR*, LytR* GraR* and VraR*
