# Supplementary material for: Systematic Reconstruction of the Complete Two-Component Sensorial Network in Staphylococcus aureus
Source: mSystems. 2020 Aug 18;5(4):e00511-20. doi: 10.1128/mSystems.00511-20 (PMC7438023; doi:10.1128/mSystems.00511-20)
Supplement: TABLE S2 [file mSystems.00511-20-st002.docx]

**Table S2 Genes regulated uniformly by multiple TCS.**

| **Locus tag** | | **Gene name** | **Up-regulated in TCS** | **Down-regulated in TCS** |
| --- | --- | --- | --- | --- |
| **Up-regulated genes** | | | | |
| MW_RS00815 | MW0155 |  | SaeR, PhoP, AirR, VraR, AgrA, KdpE, HssR, NreC, BraR |  |
| MW_RS01140 | MW0217 | *ldhA* | SaeR, Tcs7R, ArlR, SrrB, PhoP, AirR, VraR, AgrA, KdpE, HssR, NreC, BraR |  |
| MW_RS01485 | MW0282 |  | SaeR, Tcs7R, ArlR, SrrB, PhoP, AirR, VraR, AgrA, KdpE, HssR, NreC, BraR |  |
| MW_RS02155 | MW0407 | *ndhF* | SaeR, Tcs7R, ArlR, SrrB, PhoP, AirR, VraR, AgrA, KdpE, HssR, NreC, BraR |  |
| MW_RS02160 | MW0408 |  | SrrB, PhoP, AirR, VraR, AgrA, KdpE, HssR, NreC, BraR |  |
| MW_RS03155 | MW0577 |  | PhoP, AirR, VraR, AgrA, KdpE, HssR, NreC, BraR |  |
| MW_RS04000 | MW0733 | *gapR* | SaeR, ArlR, SrrB, PhoP, AirR, VraR, AgrA, KdpE, HssR, NreC, BraR |  |
| MW_RS05530 | MW1030 | *sdhC* | PhoP, AirR, VraR, AgrA, KdpE, HssR, NreC, BraR |  |
| MW_RS07730 | MW1444 |  | SaeR, ArlR, PhoP, VraR, AgrA, KdpE, HssR, NreC, |  |
| MW_RS08670 | MW1625 | *lysP* | SrrB, PhoP, VraR, AgrA, KdpE, HssR, NreC, BraR |  |
| MW_RS12655 | N/A |  | PhoP, AirR, VraR, AgrA, KdpE, HssR, NreC, BraR |  |
| MW_RS12660 | MW2338 |  | WalR, PhoP, AirR, VraR, AgrA, KdpE, HssR, NrCe, BraR |  |
| MW_RS13050 | MW2405 |  | SaeR, ArlR, SrrB, PhoP, VraR, AgrA, KdpE, HssR, NreC, BraR |  |
| **Down-regulated genes** | | | | |
| MW_RS00460 | MW0088 | *sirA* |  | WalR, SrrB, PhoP, AirR, VraR, AgrA, KdpE, HssR, NreC, BraR |
| MW_RS00960 | MW0184 | *rlp* |  | ArlR, SrrB, PhoP, AirR, KdpE, HssR, NreC, BraR |
| MW_RS01680 | MW0319 |  |  | SaeR, ArlR, SrrB, PhoP, AgrA, KdpE, HssR, NreC, BraR |
| MW_RS02205 | MW0416 | *metN1* |  | SaeR, Tcs7R, ArlR, SrrB, PhoP, AirR, VraR, AgrA, KdpE, HssR, NreC, BraR |
| MW_RS02210 | MW0417 |  |  | SaeR, ArRl, SrrB, PhoP, AirR, VraR, AgrA, KdpE, HssR, NreC, BraR |
| MW_RS02215 | MW0418 |  |  | SaeR, ArlR, SrrB, PhoP, AirR, KdEp, HssR, NreC |
| MW_RS03315 | MW0609 | *fhuC* |  | SrrB, AirR, VraR, AgrA, KdpE, HssR, NreC, BraR |
| MW_RS04285 | MW0790 | *metN2* |  | SaeR, ArlR, SrrB, PhoP, AgrA, KdpE, HssR, NreC, BraR |
| MW_RS05095 | MW0948 | *purK* |  | GraR, SaeR,Tcs7R, ArlR, SrrB, PhoP, AirR, VraR, AgrA, KdpE, HssR, NreC, BraR |
| MW_RS05100 | MW0949 | *purC* |  | SaeR, Tcs7R, ArlR, SrrB, PhoP, AirR, AgrA, KdpE, HssR, NreC, BraR |
| MW_RS05105 | MW0950 | *purS* |  | SaeR, Tcs7R, ArlR, SrrB, PhoP, AirR, VraR, AgrA, KdpE, HssR, NreC, BraR |
| MW_RS05110 | MW0951 | *purQ* |  | SaeR, ArlR, SrrB, PhoP, AirR, AgrA, KdpE, HssR, NreC, BraR |
| MW_RS05115 | MW0952 | *purL* |  | SaeR, ArlR, SrrB, PhoP, AirR, AgrA, KdpE, HssR, NreC, BraR |
| MW_RS05440 | MW1013 | *isdC* |  | SrrB, AirR, VraR, AgrA, KdpE, HssR, NreC, BraR |
| MW_RS05445 | MW1014 | *isdD* |  | SrrB, PhoP, AirR, VraR, AgrA, KdpE, HssR, NreC, BraR |
| MW_RS11450 | MW2103 |  |  | WalR, SrrB, AirR, VraR, AgrA, KdpE, HssR, NreC, BraR |
| MW_RS11965 | MW2202 | *fhuD2* |  | SrrA, PhoP, VraR, AgrA, KdpE, HssR, NreC, BraR |
