# Supplementary material for: Systematic Reconstruction of the Complete Two-Component Sensorial Network in Staphylococcus aureus
Source: mSystems. 2020 Aug 18;5(4):e00511-20. doi: 10.1128/mSystems.00511-20 (PMC7438023; doi:10.1128/mSystems.00511-20)
Supplement: TABLE S3 [file mSystems.00511-20-st003.docx]

**Table S3 Genes affected differentially by multiple TCS.**

| **Locus tag** | | **Gene name** | **Up-regulated in TCS** | **Down-regulated in TCS** |
| --- | --- | --- | --- | --- |
| **Genes** | | | | |
| MW_RS00125 | MW0023 |  | PhoP | AirR, BraR |
| MW_RS00385 | MW0073 |  | KdpE | BraR |
| MW_RS00390 | MW0074 |  | KdpE | BraR |
| MW_RS00565 | MW0108 |  | HssR, NreC, BraR | WalR |
| MW_RS01335 | MW0252 | *lytM* | WalR | KdpE |
| MW_RS01495 | MW0284 |  | SrrA | PhoP, AirR, HssR, NreC |
| MW_RS01565 | MW0297 | *lip2 geh* | WalR, SaeR, ArlR, PhoP, KdpE | SrrA, AirR, |
| MW_RS02095 | MW0396 |  | SaeR | PhoP |
| MW_RS02220 | MW0419 | *sle1 aaa* | WalR | AirR, AgrA, HssR, NreC |
| MW_RS02750 | MW0498 | *rpoC* | WalR | BraR |
| MW_RS02845 | MW0517 | *sdrD* | WalR | AirR |
| MW_RS03410 | MW0627 |  | WalR | VraR, AgrA, KdpE, HssR, NreC |
| MW_RS03910 | MW0717 |  | WalR | ArlR, AirR, KdpE |
| MW_RS06070 | MW1130 | *lytN* | KdpE | ArlR |
| MW_RS06075 | MW1131 | *fmhC* | KdpE | ArlR |
| MW_RS06280 | MW1170 |  | SaeR | WalR |
| MW_RS06290 | MW1172 |  | AirR | BraR |
| MW_RS06300 | MW1174 |  | AirR | PhoP |
| MW_RS06710 | MW1249 | *msrR* | AirR | KdpE |
| MW_RS06875 | MW1281 | *lysC* | KdpE, BraR | SrrA |
| MW_RS08040 | MW1503 | *rpmG1* | WalR | AirR, VraR, KdpE, NreC |
| MW_RS08535 | MW1600 |  | AirR, VraR | PhoP, AgrA, KdpE, HssR, NreC |
| MW_RS09085 | MW1699 |  | WalR, PhoP | ArlR |
| MW_RS09550 | MW1782 | *prsA* | AirR, VraR | WalR |
| MW_RS10640 | MW1956 |  | WalR | AgrA, HssR, NreC |
| MW_RS11055 | MW2028 | *atpG* | SrrA | BraR |
| MW_RS11060 | MW2029 | *atpA* | SrrA | BraR |
| MW_RS11065 | MW2030 | *atpH* | SrrA | BraR |
| MW_RS11070 | MW2031 | *atpF* | SrrA | BraR |
| MW_RS11320 | MW2086 | truncated *fmtB* | WalR | ArlR, AirR, BraR |
| MW_RS11665 | MW2146 | *rpmJ* | WalR, PhoP | BraR |
| MW_RS11695 | MW2152 | *rpsE* | WalR, PhoP | BraR |
| MW_RS11700 | MW2153 | *rplR* | WalR, PhoP | BraR |
| MW_RS11705 | MW2154 | *rplF* | WalR, PhoP | BraR |
| MW_RS11710 | MW2155 | *rpsH* | WalR, PhoP | BraR |
| MW_RS11715 | MW2156 | *rpsZ rpsN1* | WalR, PhoP | BraR |
| MW_RS11720 | MW2157 | *rplE* | WalR, PhoP | BraR |
| MW_RS11725 | MW2158 | *rplX* | WalR, PhoP | BraR |
| MW_RS11730 | MW2159 | *rplN* | WalR. PhoP | BraR |
| MW_RS11735 | MW2160 | *rpsQ* | WalR, PhoP | BraR |
| MW_RS11740 | MW2161 | *rpmC* | WalR, PhoP | BraR |
| MW_RS11745 | MW2162 | *rplP* | WalR, PhoP | BraR |
| MW_RS11750 | MW2163 | *rpsC* | WalR, PhoP | BraR |
| MW_RS11755 | MW2164 | *rplV* | WalR, PhoP | BraR |
| MW_RS11760 | MW2165 | *rpsS* | WalR, PhoP | BraR |
| MW_RS12000 | MW2210 | *ureF* | AirR | WalR, ArlR |
| MW_RS12010 | MW2212 | *ureD* | AirR | WalR, ArlR |
| MW_RS12040 | MW2217 | *ssaA2* | WalR, GraR | VraR, KdpE |
| MW_RS12065 | MW2222 | *ssaA* | WalR | TCS7, SrrA, VraR, AgrA, KdpE, HssR, NreC |
| MW_RS12230 | MW2255 | *lyrA* | WalR | VraR, AgrA, HssR, NreC |
| MW_RS13195 | MW2433 |  | ArlR | PhoP, AirR, KdpE |
| MW_RS13200 | MW2434 |  | ArlR | AirR |
| MW_RS13345 | MW2462 | *cidA* | SaeR, PhoP, AgrA, KdpE, HssR, NreC | SrrA |
| MW_RS13360 | MW2465 |  | WalR | SaeR, TCS7, PhoP, VraR, AgrA, KdpE, HssR, NreC |
| MW_RS14030 | MW2585 | *icaR* | ArlR, HssR, NreC, BraR | WalR |
| MW_RS14245 | MW2628 | *rsmG* | WalR | BraR |
| MW_RS14865 | N/A | N/A | WalR | PhoP, AirR |
| **Proteins** | | | | |
| MW_RS01495 | MW0284 |  | SrrA | AirR |
| MW_RS04430 | MW0818 |  | AirR | WalR |
| MW_RS07055 | MW1314 | msrA2 msrA | AirR | SrrA, |
| MW_RS11320 | MW2086 | truncated fmtB | WalR | ArlR |
