# Supplementary material for: Systematic Reconstruction of the Complete Two-Component Sensorial Network in Staphylococcus aureus
Source: mSystems. 2020 Aug 18;5(4):e00511-20. doi: 10.1128/mSystems.00511-20 (PMC7438023; doi:10.1128/mSystems.00511-20)
Supplement: TABLE S4 [file mSystems.00511-20-st004.docx]

Table S4 Strains, plasmids and oligonucleotides used in this study.

| **Strain name** | **Relevant characteristic(s)** | **MIC†** | **Source or reference** |
| --- | --- | --- | --- |
| MW2 | Community-acquired strain of MRSA, which was isolated in 1998 in North Dakota, USA | 3566 | (1) |
| ΔXV | MW2 ΔXIV Δ*srrBA* | 2961 | (2) |
| MW2 pCN51 | MW2 carrying pCN51 empty plasmid | 4810 | (2) |
| ΔXV pCN51 | MW2 ΔXV carrying pCN51 empty plasmid | 4671 | (2) |
| ΔXV p*saeRS* | MW2 ΔXV carrying pCN51::*saeRS* plasmid | 5233 | (2) |
| ΔXV p*nreCB* | MW2 ΔXV carrying pCN51::*nreCB* plasmid | 4536 | (2) |
| ΔXV p*braRS* | MW2 ΔXV carrying pCN51::*braRS* plasmid | 5238 | (2) |
| ΔXV p*nreCB-*3xFLAG | MW2 ΔXV carrying pCN51::*nreCB*-3xFLAG plasmid | 5900 | This study |
| MW2 pRMC2 | MW2 carrying pRMC2 empty plasmid | 6870 | This study |
| ΔXV pRMC2 | MW2 ΔXV carrying pRMC2 empty plasmid | 6871 | This study |
| ΔXV p*walR** | MW2 ΔXV carrying pRMC2::*walR* plasmid (pAH135) | 6872 | This study |
| ΔXV p*hptR** | MW2 ΔXV carrying pRMC2::*hptR* plasmid (pAH135) | 6873 | This study |
| ΔXV p*lytR** | MW2 ΔXV carrying pRMC2::*lytR* plasmid (pAH137) | 6874 | This study |
| ΔXV p*graR** | MW2 ΔXV carrying pRMC2::*graR* plasmid (pAH138) | 6875 | This study |
| ΔXV p*saeR** | MW2 ΔXV carrying pRMC2::*saeR* plasmid (pAH139) | 6876 | This study |
| ΔXV p*tcs7R** | MW2 ΔXV carrying pRMC2::*tcs7R* plasmid (pAH140) | 6877 | This study |
| ΔXV p*arlR** | MW2 ΔXV carrying pRMC2::*arlR* plasmid (pAH141) | 6878 | This study |
| ΔXV p*srrA** | MW2 ΔXV carrying pRMC2::*srrA* plasmid (pAH142) | 6879 | This study |
| ΔXV p*phoP** | MW2 ΔXV carrying pRMC2::*phoP* plasmid (pAH143) | 6880 | This study |
| ΔXV p*airR** | MW2 ΔXV carrying pRMC2::*airR* plasmid (pAH144) | 6881 | This study |
| ΔXV p*vraR** | MW2 ΔXV carrying pRMC2::*vraR* plasmid (pAH145) | 6882 | This study |
| ΔXV p*agrA** | MW2 ΔXV carrying pRMC2::*agrA* plasmid (pAH146) | 6883 | This study |
| ΔXV p*kdpE** | MW2 ΔXV carrying pRMC2::*kdpE* plasmid (pAH147) | 6884 | This study |
| ΔXV p*hssR** | MW2 ΔXV carrying pRMC2::*hssR* plasmid (pAH148) | 6885 | This study |
| ΔXV p*nreC** | MW2 ΔXV carrying pRMC2::*nreC* plasmid (pAH149) | 6886 | This study |
| ΔXV p*braR** | MW2 ΔXV carrying pRMC2::*braR* plasmid (pAH150) | 6887 | This study |
| ΔXV p*nreC*-3xFLAG | MW2 ΔXV carrying pRMC2::*nreC*-3xFLAG plasmid | 7678 | This study |
| MW2 pRMC2 +pCN52 | MW2 carrying pRMC2 and pCN52 empty plasmids | 7354 | This study |
| ΔXV pRMC2 +pCN52 | MW2 ΔXV carrying pRMC2 and pCN52 empty plasmids | 7355 | This study |
| MW2 Δ*hpt* | MW2 Δ*hptSR* | 4032 | (2) |
| MW2 Δ*lyt* | MW2 Δ*lytSR* | 2964 | (2) |
| MW2 Δ*gra* | MW2 Δ*graRS* | 11 | (2) |
| MW2 Δ*sae* | MW2 Δ*saeSR* | 2965 | (2) |
| MW2 Δ*tcs7* | MW2 Δ*tcs7SR* | 4033 | (2) |
| MW2 Δ*arl* | MW2 Δ*arlSR* | 4043 | (2) |
| MW2 Δ*srr* | MW2 Δ*srrBA* | 2966 | (2) |
| MW2 Δ*pho* | MW2 Δ*phoRP* | 4035 | (2) |
| MW2 Δ*air* | MW2 Δ*airRS* | 3670 | (2) |
| MW2 Δ*vra* | MW2 Δ*vraRS* | 4036 | (2) |
| MW2 Δ*agr* | ΔXV Δ*agrCA* | 4037 | (2) |
| MW2 Δ*kdp* | MW2 Δ*kdpDE* | 4038 | (2) |
| MW2 Δ*hss* | MW2 Δ*hssRS* | 2979 | (2) |
| MW2 Δ*nre* | MW2 Δ*nreCB* | 2967 | (2) |
| MW2 Δ*bra* | MW2 Δ*braSR* | 4039 | (2) |
| ΔXV p*walR** + pPatl::GFP | MW2 ΔXV carrying pRMC2::*walR and* pCN52::P*atl*::*gfp* plasmids | 7762 | This study |
| ΔXV p*hptR** +pPuhpT::GFP | MW2 ΔXV carrying pRMC2::*hptR* and pCN52::P*uhpT*::*gfp* plasmids | 7389 | This study |
| ΔXV p*lytR** + pPlrgA::GFP | MW2 ΔXV carrying pRMC2::*lytR* and pCN52::P*lrgA*::*gfp* plasmids | 7391 | This study |
| ΔXV p*graR** + pPmprF::GFP | MW2 ΔXV carrying pRMC2::*graR* and pCN52::P*mprF*::*gfp* plasmids | 7393 | This study |
| ΔXV p*saeR** + pPefb::GFP | MW2 ΔXV carrying pRMC2::*saeR* and pCN52::P*efb*::*gfp* plasmids | 7357 | This study |
| ΔXV p*tcs7R** + pPMW1206::GFP | MW2 ΔXV carrying pRMC2::*tcs7R* and pCN52::P*MW1206*::*gfp* plasmids | 7833 | This study |
| ΔXV p*arlR** + pPmgrA::GFP | MW2 ΔXV carrying pRMC2::*arlR* and pCN52::P*mgrA*::*gfp* plasmids | 7359 | This study |
| ΔXV p*srrA** + pPqox::GFP | MW2 ΔXV carrying pRMC2::*srrA* and pCN52::P*qox*::*gfp* plasmids | 7362 | This study |
| ΔXV p*phoP** + pPpstS::GFP | MW2 ΔXV carrying pRMC2::*phoP* and pCN52::P*pstS*::*gfp* plasmids | 7486 | This study |
| ΔXV p*airR** + pPMW2540::GFP | MW2 ΔXV carrying pRMC2::*airR* and pCN52::P*MW2540*::*gfp* plasmids | 7536 | This study |
| ΔXV p*agrA** + pPMW1056::GFP | MW2 ΔXV carrying pRMC2::*agrA* and pCN52::P*MW1056*::*gfp* plasmids | 7545 | This study |
| ΔXV p*kdpE** + pPcap8A::GFP | MW2 ΔXV carrying pRMC2::*kdpE* and pCN52::P*cap8A*::*gfp* plasmids | 7487 | This study |
| ΔXV p*hssR** + pPhrtA::GFP | MW2 ΔXV carrying pRMC2::*hssR* and pCN52::P*hrtA*::*gfp* plasmids | 7661 | This study |
| ΔXV p*nreC* *+ pPnarG::GFP | MW2 ΔXV carrying pRMC2::*nreC* and pCN52::P*narG*::*gfp* plasmids | 7539 | This study |
| ΔXV p*braR** + pPctsR::GFP | MW2 ΔXV carrying pRMC2::*braR* and pCN52::P*ctsR*::*gfp* plasmids | 7488 | This study |
| MW2 pRMC2 +pPefb::GFP | MW2 carrying pRMC2 empty plasmid and pCN52::P*efb*::*gfp* plasmid | 7801 | This study |
| ΔXV pRMC2 + pPefb::GFP | MW2 ΔXV carrying pRMC2empty plasmid and pCN52::P*efb*::*gfp* plasmid | 7381 | This study |
| ΔXV p*walR** + pPefb::GFP | MW2 ΔXV carrying pRMC2::*walR* and pCN52::P*efb*::*gfp* plasmids | 7802 | This study |
| ΔXV p*hptR** +pPefb::GFP | MW2 ΔXV carrying pRMC2::*hptR* and pCN52::P*efb*::*gfp* plasmids | 7803 | This study |
| ΔXV p*lytR** + pPefb::GFP | MW2 ΔXV carrying pRMC2::*lytR* and pCN52::P*efb*::*gfp* plasmids | 7804 | This study |
| ΔXV p*graR** + pPefb::GFP | MW2 ΔXV carrying pRMC2::*graR* and pCN52::P*efb*::*gfp* plasmids | 7805 | This study |
| ΔXV p*tcs7R** + pPefb::GFP | MW2 ΔXV carrying pRMC2::*tcs7R* and pCN52::P*efb*::*gfp* plasmids | 7806 | This study |
| ΔXV p*arlR** + pPefb::GFP | MW2 ΔXV carrying pRMC2::*arlR* and pCN52::P*efb*::*gfp* plasmids | 7807 | This study |
| ΔXV p*srrA** + pPefb::GFP | MW2 ΔXV carrying pRMC2::*srrA* and pCN52::P*efb*::*gfp* plasmids | 7808 | This study |
| ΔXV p*phoP** + pPefb::GFP | MW2 ΔXV carrying pRMC2::*phoP* and pCN52::P*efb*::*gfp* plasmids | 7809 | This study |
| ΔXV p*airR** + pPefb::GFP | MW2 ΔXV carrying pRMC2::*airR* and pCN52::P*efb*::*gfp* plasmids | 7810 | This study |
| ΔXV p*vraR** + pPefb::GFP | MW2 ΔXV carrying pRMC2::*vraR* and pCN52::P*efb*::*gfp* plasmids | 7811 | This study |
| ΔXV p*agrA** + pPefb::GFP | MW2 ΔXV carrying pRMC2::*agrA* and pCN52::P*efb*::*gfp* plasmids | 7812 | This study |
| ΔXV p*kdpE** + pefb::GFP | MW2 ΔXV carrying pRMC2::*kdpE* and pCN52::P*efb*::*gfp* plasmids | 7813 | This study |
| ΔXV p*hssR** + pPefb::GFP | MW2 ΔXV carrying pRMC2::*hssR* and pCN52::P*efb*::*gfp* plasmids | 7814 | This study |
| ΔXV p*nreC** + pPefb::GFP | MW2 ΔXV carrying pRMC2::*nreC* and pCN52::P*efb*::*gfp* plasmids | 7815 | This study |
| ΔXV p*braR** + pPefb::GFP | MW2 ΔXV carrying pRMC2::*braR* and pCN52::P*efb*::*gfp* plasmids | 7816 | This study |
| MW2 +pPefb::GFP | MW2 carrying pCN52::P*efb*::*gfp* plasmid | 6131 | This study |
| ΔXV + pPefb::GFP | ΔXV carrying pCN52::P*efb*::gfp plasmid | 6132 | This study |
| MW2 Δ*hpt* +pPefb::GFP | MW2 Δ*hptSR* carrying pCN52::P*efb*::*gfp* plasmid | 6149 | This study |
| MW2 Δ*lyt* + pPefb::GFP | MW2 Δ*lytSR* carrying pCN52::P*efb*::*gfp* plasmid | 6150 | This study |
| MW2 Δ*gra* + pPefb::GFP | MW2 Δ*graRS* carrying pCN52::P*efb*::*gfp* plasmid | 6151 | This study |
| MW2 Δ*sae* + pPefb::GFP | MW2 Δ*saeSR* carrying pCN52::P*efb*::*gfp* plasmid | 6133 | This study |
| MW2 Δ*tcs7* + pPefb::GFP | MW2 Δ*tcs7SR* carrying pCN52::P*efb*::*gfp* plasmid | 6152 | This study |
| MW2 Δ*arl* + pPefb::GFP | MW2 Δ*arlSR* carrying pCN52::P*efb*::*gfp* plasmid | 6153 | This study |
| MW2 Δ*srr* + pPefb::GFP | MW2 Δ*srrBA* carrying pCN52::P*efb*::*gfp* plasmid | 6154 | This study |
| MW2 Δ*pho* + pPefb::GFP | MW2 Δp*hoRP* carrying pCN52::P*efb*::*gfp* plasmid | 6155 | This study |
| MW2 Δ*air* + pPefb::GFP | MW2 Δ*airRS* carrying pCN52::P*efb*::*gfp* plasmid | 6156 | This study |
| MW2 Δ*vra* + pPefb::GFP | MW2 Δ*vraRS* carrying pCN52::P*efb*::*gfp* plasmid | 6157 | This study |
| MW2 Δ*agr* + pPefb::GFP | MW2 Δ*agrCA* carrying pCN52::P*efb*::*gfp* plasmid | 6158 | This study |
| MW2 Δ*kdp* + pPefb::GFP | MW2 Δ*kdpDE* carrying pCN52::P*efb*::*gfp* plasmid | 6159 | This study |
| MW2 Δ*hss* + pPefb::GFP | MW2 Δ*hssRS* carrying pCN52::P*efb*::*gfp* plasmid | 6160 | This study |
| MW2 Δ*nre* + pPefb::GFP | MW2 Δ*nreCB* carrying pCN52::P*efb*::*gfp* plasmid | 6161 | This study |
| MW2 Δ*bra* + pPefb::GFP | MW2 Δ*braSR* carrying pCN52::P*efb*::*gfp* plasmid | 6162 | This study |
| MW2 pRMC2 +pPmgrA::GFP | MW2 carrying pRMC2 empty plasmid and pCN52::P*mgrA*::*gfp* plasmid | 7531 | This study |
| ΔXV pRMC2 + pPmgrA::GFP | MW2 ΔXV carrying pRMC2 empty plasmid and pCN52::P*mgrA*::*gfp* plasmid | 7360 | This study |
| ΔXV p*walR** + pPmgrA::GFP | MW2 ΔXV carrying pRMC2::*walR* and pCN52::P*mgrA*::*gfp* plasmids | 7530 | This study |
| ΔXV p*hptR** +pPmgrA::GFP | MW2 ΔXV carrying pRMC2::*hptR* and pCN52::P*mgrA*::*gfp* plasmids | 7490 | This study |
| ΔXV p*lytR** + pPmgrA::GFP | MW2 ΔXV carrying pRMC2::*lytR* and pCN52::P*mgrA*::*gfp* plasmids | 7491 | This study |
| ΔXV p*graR** + pPmgrA::GFP | MW2 ΔXV carrying pRMC2::*graR* and pCN52::P*mgrA*::*gfp* plasmids | 7492 | This study |
| ΔXV p*saeR** + pPmgrA::GFP | MW2 ΔXV carrying pRMC2::*saeR* and pCN52::P*mgrA*::*gfp* plasmids | 7493 | This study |
| ΔXV p*tcs7R** + pPmgrA::GFP | MW2 ΔXV carrying pRMC2::*tcs7R* and pCN52::P*mgrA*::*gfp* plasmids | 7494 | This study |
| ΔXV p*srrA** + pPmgrA::GFP | MW2 ΔXV carrying pRMC2::*srrA* and pCN52::P*mgrA*::*gfp* plasmids | 7495 | This study |
| ΔXV p*phoP** + pPmgrA::GFP | MW2 ΔXV carrying pRMC2::*phP* and pCN52::P*mgrA*::*gfp* plasmids | 7496 | This study |
| ΔXV p*airR** + pPmgrA::GFP | MW2 ΔXV carrying pRMC2::*airR* and pCN52::P*mgrA*::*gfp* plasmids | 7497 | This study |
| ΔXV p*vraR** + pPmgrA::GFP | MW2 ΔXV carrying pRMC2::*vraR* and pCN52::P*mgrA*::*gfp* plasmids | 7498 | This study |
| ΔXV p*agrA** + pPmgrA::GFP | MW2 ΔXV carrying pRMC2::*agrA* and pCN52::P*mgrA*::*gfp* plasmids | 7499 | This study |
| ΔXV p*kdpE** + pPmgrA::GFP | MW2 ΔXV carrying pRMC2::*kdpE* and pCN52::P*mgrA*::*gfp* plasmids | 7500 | This study |
| ΔXV p*hssR** + pPmgrA::GFP | MW2 ΔXV carrying pRMC2::*hssR* and pCN52::P*mgrA*::*gfp* plasmids | 7501 | This study |
| ΔXV p*nreC** + pPmgrA::GFP | MW2 ΔXV carrying pRMC2::*nreC* and pCN52::P*mgrA*::*gfp* plasmids | 7502 | This study |
| ΔXV p*braR** + pPmgrA::GFP | MW2 ΔXV carrying pRMC2::*braR* and pCN52::P*mgrA*::*gfp* plasmids | 7503 | This study |
| MW2 + pPmgrA::GFP | MW2 carrying pCN52::P*mgrA*::*gfp* plasmid | 5990 | (3) |
| ΔXV + pPmgrA::GFP | ΔXV carrying pCN52::P*mgrA*::gfp plasmid | 6049 | This study |
| MW2 Δ*hpt* + pPmgrA::GFP | MW2 Δ*hptSR* carrying pCN52::P*mgrA*::*gfp* plasmid | 6066 | This study |
| MW2 Δ*lyt* + pPmgrA::GFP | MW2 Δ*lytSR* carrying pCN52::P*mgrA*::*gfp* plasmid | 6067 | This study |
| MW2 Δ*gra* + pPmgrA::GFP | MW2 Δ*graRS* carrying pCN52::P*mgrA*::*gfp* plasmid | 6068 | This study |
| MW2 Δ*sae* + pPmgrA::GFP | MW2 Δ*saeSR* carrying pCN52::P*mgrA*::*gfp* plasmid | 6069 | This study |
| MW2 Δ*tcs7* + pPmgrA::GFP | MW2 Δ*tcs7SR* carrying pCN52::P*mgrA*::*gfp* plasmid | 6070 | This study |
| MW2 Δ*arl* + pPmgrA::GFP | MW2 Δ*arlSR* carrying pCN52::P*mgrA*::*gfp* plasmid | 5991 | (3) |
| MW2 Δ*srr* + pPmgrA::GFP | MW2 Δ*srrBA* carrying pCN52::P*mgrA*::*gfp* plasmid | 6071 | This study |
| MW2 Δ*pho* + pPmgrA::GFP | MW2 Δp*hoRP* carrying pCN52::P*mgrA*::*gfp* plasmid | 6072 | This study |
| MW2 Δ*air* + pPmgrA::GFP | MW2 Δ*airRS* carrying pCN52::P*mgrA*::*gfp* plasmid | 6073 | This study |
| MW2 Δ*vra* + pPmgrA::GFP | MW2 Δ*vraRS* carrying pCN52::P*mgrA*::*gfp* plasmid | 6074 | This study |
| MW2 Δ*agr* + pPmgrA::GFP | MW2 Δ*agrCA* carrying pCN52::P*mgrA*::*gfp* plasmid | 6075 | This study |
| MW2 Δ*kdp* + pPmgrA::GFP | MW2 Δ*kdpDE* carrying pCN52::P*mgrA*::*gfp* plasmid | 6076 | This study |
| MW2 Δ*hss* + pPmgrA::GFP | MW2 Δ*hssRS* carrying pCN52::P*mgrA*::*gfp* plasmid | 6077 | This study |
| MW2 Δ*nre* + pPmgrA::GFP | MW2 Δ*nreCB* carrying pCN52::P*mgrA*::*gfp* plasmid | 6078 | This study |
| MW2 Δ*bra* + pPmgrA::GFP | MW2 Δ*braSR* carrying pCN52::P*mgrA*::*gfp* plasmid | 6079 | This study |
| MW2 pRMC2 +pPqox::GFP | MW2 carrying pRMC2 empty plasmid and pCN52::P*qox*::*gfp* plasmid | 7817 | This study |
| ΔXV pRMC2+ pPqox::GFP | MW2 ΔXV carrying pRMC2 empty plasmid and pCN52::P*qox*::*gfp* plasmid | 7382 | This study |
| ΔXV p*walR** + pPqox::GFP | MW2 ΔXV carrying pRMC2::*walR* and pCN52::P*qox*::*gfp* plasmids | 7818 | This study |
| ΔXV p*hptR** +pPqox::GFP | MW2 ΔXV carrying pRMC2::*hptR* and pCN52::P*qox*::*gfp* plasmids | 7819 | This study |
| ΔXV p*lytR** + pPqox::GFP | MW2 ΔXV carrying pRMC2::*lytR* and pCN52::P*qox*::*gfp* plasmids | 7820 | This study |
| ΔXV p*graR** + pPqox::GFP | MW2 ΔXV carrying pRMC2::*graR* and pCN52::P*qox*::*gfp* plasmids | 7821 | This study |
| ΔXV p*saeR** + pPqox::GFP | MW2 ΔXV carrying pRMC2::*saeR* and pCN52::P*qox*::*gfp* plasmids | 7822 | This study |
| ΔXV p*tcs7R** + pPqox::GFP | MW2 ΔXV carrying pRMC2::*tcs7R* and pCN52::P*qox*::*gfp* plasmids | 7823 | This study |
| ΔXV p*arlR** + pPqox::GFP | MW2 ΔXV carrying pRMC2::*arlR* and pCN52::P*qox*::*gfp* plasmids | 7824 | This study |
| ΔXV p*phoP** + pPqox::GFP | MW2 ΔXV carrying pRMC2::*phoP* and pCN52::P*qox*::*gfp* plasmids | 7825 | This study |
| ΔXV p*airR** + pPqox::GFP | MW2 ΔXV carrying pRMC2::*airR* and pCN52::P*qox*::*gfp* plasmids | 7826 | This study |
| ΔXV p*vraR** + pPqox::GFP | MW2 ΔXV carrying pRMC2::*vraR* and pCN52::P*qox*::*gfp* plasmids | 7827 | This study |
| ΔXV p*agrA** + pPqox::GFP | MW2 ΔXV carrying pRMC2::*agrA* and pCN52::P*qox*::*gfp* plasmids | 7828 | This study |
| ΔXV p*kdpE** + pqox::GFP | MW2 ΔXV carrying pRMC2::*kdpE* and pCN52::P*qox*::*gfp* plasmids | 7829 | This study |
| ΔXV p*hssR** + pPqox::GFP | MW2 ΔXV carrying pRMC2::*hssR* and pCN52::P*qox*::*gfp* plasmids | 7830 | This study |
| ΔXV p*nreC** + pPqox::GFP | MW2 ΔXV carrying pRMC2::*nreC* and pCN52::P*qox*::*gfp* plasmids | 7831 | This study |
| ΔXV p*braR** + pPqox::GFP | MW2 ΔXV carrying pRMC2::*braR* and pCN52::P*qox*::*gfp* plasmids | 7832 | This study |
| MW2 +pPqox::GFP | MW2 carrying pCN52::Pqox::*gfp* plasmid | 6178 | This study |
| ΔXV + pPqox::GFP | ΔXV carrying pCN52::P*qox::gfp* plasmid | 6179 | This study |
| MW2 Δ*hpt* +pPqox::GFP | MW2 Δ*hptSR* carrying pCN52::P*qox*::*gfp* plasmid | 6180 | This study |
| MW2 Δ*lyt* + pPqox::GFP | MW2 Δ*lytSR* carrying pCN52::P*qox*::*gfp* plasmid | 6181 | This study |
| MW2 Δ*gra* + pPqox::GFP | MW2 Δ*graRS* carrying pCN52::P*qox*::*gfp* plasmid | 6182 | This study |
| MW2 Δ*sae* + pPqox::GFP | MW2 Δ*saeSR* carrying pCN52::P*qox*::*gfp* plasmid | 6183 | This study |
| MW2 Δ*tcs7* + pPqox::GFP | MW2 Δ*tcs7SR* carrying pCN52::P*qox*::*gfp* plasmid | 6184 | This study |
| MW2 Δ*arl* + pPqox::GFP | MW2 Δ*arlSR* carrying pCN52::P*qox*::*gfp* plasmid | 6185 | This study |
| MW2 Δ*srr* + pPqox::GFP | MW2 Δ*srrBA* carrying pCN52::P*qox*::*gfp* plasmid | 6186 | This study |
| MW2 Δ*pho* + pPqox::GFP | MW2 Δp*hoRP* carrying pCN52::P*qox*::*gfp* plasmid | 6187 | This study |
| MW2 Δ*air* + pPqox::GFP | MW2 Δ*airRS* carrying pCN52::P*qox*::*gfp* plasmid | 6188 | This study |
| MW2 Δ*vra* + pPqox::GFP | MW2 Δ*vraRS* carrying pCN52::P*qox*::*gfp* plasmid | 6189 | This study |
| MW2 Δ*agr* + pPqox::GFP | MW2 Δ*agrCA* carrying pCN52::P*qox*::*gfp* plasmid | 6190 | This study |
| MW2 Δ*kdp* + pPqox::GFP | MW2 Δ*kdpDE* carrying pCN52::P*qox*::*gfp* plasmid | 6191 | This study |
| MW2 Δ*hss* + pPqox::GFP | MW2 Δ*hssRS* carrying pCN52::P*qox*::*gfp* plasmid | 6192 | This study |
| MW2 Δ*nre* + pPqox::GFP | MW2 Δ*nreCB* carrying pCN52::P*qox*::*gfp* plasmid | 6193 | This study |
| MW2 Δ*bra* + pPqox::GFP | MW2 Δ*braSR* carrying pCN52::P*qox*::*gfp* plasmid | 6194 | This study |

† Microbial Pathogenesis Laboratory collection number

| **Plasmid name** | **Description** | **Source or reference** |
| --- | --- | --- |
| pCN51 | *E. coli* – *S. aureus* shuttle vector to express genes under the control of Pcad cadmium inducible promoter. EmR | (4) |
| pCN51::*saeRS* | pCN51 plasmid expressing *saeRS* genes | (2) |
| pCN51::*nreCB* | pCN51 plasmid expressing *nreCB* genes | (2) |
| pCN51::*braRS* | pCN51 plasmid expressing *braRS* genes | (2) |
| pCN51::*nreCB*-3xFLAG | pCN51 plasmid expressing *nreCB* genes with *nreC* expressing the 3xflag epitope at the C-terminus. | This study |
| pRMC2 | anhydrotetracycline-inducible expression plasmid | (5) |
| pRMC2::*nreC*-3xFLAG | pRMCC2 plasmid expressing *nreC** gene with *nreC** expressing the 3xflag epitope at the C-terminus | This study |
| pAH0135 | pRMC2 plasmid expressing the phosphomimetic form of *walR* | This study |
| pAH0136 | pRMC2 plasmid expressing the phosphomimetic form of *hptR* | This study |
| pAH0137 | pRMC2 plasmid expressing the phosphomimetic form of *lytR* | This study |
| pAH0138 | pRMC2 plasmid expressing the phosphomimetic form of *graR* | This study |
| pAH0139 | pRMC2 plasmid expressing the phosphomimetic form of *saeR* | This study |
| pAH0140 | pRMC2 plasmid expressing the phosphomimetic form of *tcs7R* | This study |
| pAH0141 | pRMC2 plasmid expressing the phosphomimetic form of *arlR* | This study |
| pAH0142 | pRMC2 plasmid expressing the phosphomimetic form of *srrA* | This study |
| pAH0143 | pRMC2 plasmid expressing the phosphomimetic form of *phoP* | This study |
| pAH0144 | pRMC2 plasmid expressing the phosphomimetic form of *airR* | This study |
| pAH0145 | pRMC2 plasmid expressing the phosphomimetic form of *vraR* | This study |
| pAH0146 | pRMC2 plasmid expressing the phosphomimetic form of *agrA* | This study |
| pAH0147 | pRMC2 plasmid expressing the phosphomimetic form of *kdpE* | This study |
| pAH0148 | pRMC2 plasmid expressing the phosphomimetic form of *hssR* | This study |
| pAH0149 | pRMC2 plasmid expressing the phosphomimetic form of *nreC* | This study |
| pAH0150 | pRMC2 plasmid expressing the phosphomimetic form of *braR* | This study |
| pCN52 | *E. coli* – *S. aureus* shuttle vector with promoterless *gfpmut2* fusion vector. EmR | (4) |
| pCN52::P*atl*::*gfp* plasmid | pCN52 plasmid expressing *gfpmut2* under the *atl* promoter | This study |
| pCN52::P*uhpT*::*gfp* plasmid | pCN52 plasmid expressing *gfpmut2* under the *uhpT* promoter | This study |
| pCN52::P*lrgA*::*gfp* plasmid | pCN52 plasmid expressing *gfpmut2* under the *lrgA* promoter | This study |
| **Plasmid name** | **Description** | **Source or reference** |
| pCN52::P*mprF*::*gfp* plasmid | pCN52 plasmid expressing *gfpmut2* under the *mprF* promoter | This study |
| pCN52::P*efb*::*gfp* plasmid | pCN52 plasmid expressing *gfpmut2* under the *efb* promoter | This study |
| pCN52::P*MW1206*::*gfp* plasmid | pCN52 plasmid expressing *gfpmut2* under the *MW1206* promoter | This study |
| pCN52::P*mgrA*::*gfp* plasmid | pCN52 plasmid expressing *gfpmut2* under the *mgrA* promoter | (3) |
| pCN52::P*qox*::*gfp* plasmid | pCN52 plasmid expressing *gfpmut2* under the *qox* promoter | This study |
| pCN52::P*pstS*::*gfp* plasmid | pCN52 plasmid expressing *gfpmut2* under the *pstS* promoter | This study |
| pCN52::P*MW2540*::*gfp* plasmid | pCN52 plasmid expressing *gfpmut2* under the *MW2540* promoter | This study |
| pCN52::P*MW1056*::*gfp* plasmid | pCN52 plasmid expressing *gfpmut2* under the *MW1056* promoter | This study |
| pCN52::P*cap8A*::*gfp* plasmid | pCN52 plasmid expressing *gfpmut2* under the *cap8A* promoter | This study |
| pCN52::P*hrtA:*:*gfp* plasmid | pCN52 plasmid expressing *gfpmut2* under the *hrtA* promoter | This study |
| pCN52::P*narG*::*gfp* plasmid | pCN52 plasmid expressing *gfpmut2* under the *narG* promoter | This study |
| pCN52::P*ctsR*::*gfp* plasmid | pCN52 plasmid expressing *gfpmut2* under the *ctsR* promoter | This study |

| Primer name | Sequence 5’-3’ | Plasmid name | Source or Reference |
| --- | --- | --- | --- |
| TCS phosphomimetic mutants | | | |
| MW2_walR_1_KF | CTTGAT***GGTACC*AGGAGGAAATT**ATGGCTAGAAAAGTTGTTGTAGTTG | pAH0135 | This study |
| MW2_walR_3_D52E_R* | CAGGTAACATGAT**CTC**TAGTAATACGATGTCTGGTTCTTC |  |  |
| MW2_walR_3_D52E_F* | GACATCGTATTACTA**GAG**ATCATGTTACCTGGTCGTGATG |  |  |
| MW2_walR_2_ScR | GAATTC***GAGCTC***CACTTCATTCGTTTCGACCTC |  |  |
| MW2_TCS3RR_1_KF | CTTGAT***GGTACC*AGGAGGAAATT**ATGTTTAAGGTAGTTATTTGTGATGATGAAC | pAH0136 | This study |
| MW2_TCS3RR_3_D55E_R* | GCATACGAAT**CTC**AGTAATTACTAATTCTGGCTGATGTTG |  |  |
| MW2_TCS3RR_3_D55E_F* | AATTAGTAATTACT**GAG**ATTCGTATGCCACGTAAAAATGG |  |  |
| MW2_TCS3RR_2_ScR | GAATTC***GAGCTC***GTAGGCGCCATAATCATTAGTTATGTG |  |  |
| MW2_lytR_1_KF | CTTGAT***GGTACC*AGGAGGAAATT**ATGAAAGCATTAATCATAGATGATGAG | pAH0137 | This study |
| MW2_lytR_3_D53E_R* | AATTGACCTCTAAAAATATAATGTCATATTGATTGATCAG |  |  |
| MW2_lytR_3_D53E_F* | TATATTTTTA**GAG**GTCAATTTAATGGATGAAAATGGGATC |  |  |
| MW2_lytR_2_ScR | GAATTC***GAGCTC***GGATTACTGTTAAAGTAACCCTATCGAC |  |  |
| MW2_graR_1_KF | CTTGAT***GGTACC*AGGAGGAAATT**ATGCAAATACTACTAGTAGAAGATGACAATAC | pAH0138 | This study |
| MW2_graR_3_D51E_R* | GGTAATTGAACCTCCAATATAACAATTTCAGGATTAAAAC |  |  |
| MW2_graR_3_D51E_F* | AATTGTTATATTG**GAG**GTTCAATTACCTAAATATGATGGG |  |  |
| MW2_graR_2_ScR | GAATTC***GAGCTC***GCTACCCATTTCAAATTATTCATGAG |  |  |
| MW2_saeR_1_KF | CTTGAT***GGTACC*AGGAGGAAATT**ATGACCCACTTACTGATCGTG | pAH0139 | This study |
| MW2_saeR_3_D51E_R* | TCATGATCTCAAGTACCATGATATCAATATCATTTGATAG |  |  |
| MW2_saeR_3_D51E_F* | ATATCATGGTACTT**GAG**ATCATGATGCCAGAAGTTAATGG |  |  |
| MW2_saeR_2_ScR | GAATTC***GAGCTC***GACTTCTAATTGATAACACCATTATCG |  |  |
| MW2_TCS7RR_1_KF | CTTGAT***GGTACC*AGGAGGAAATT**ATGACATCTTTAATTATTGCAGAAGATCA | pAH0140 | This study |
| MW2_TCS7RR_3_D54E_R* | GCATTTCTAT**CTC**TAAAATAACAACGTTAGGATTATATTC |  |  |
| MW2_TCS7RR_3_D54E_F* | CTAACGTTGTTATTTTA**GAG**ATAGAAATGCCAGGCATGAC |  |  |
| MW2_TCS7RR_2_ScR | GAATTC***GAGCTC***GTTTGTATTTAGATCCAGCCTTTTTC |  |  |
| MW2_arlR_1_KF | TTGAT***GGTACC*AGGAGGAAATT**ATGACGCAAATTTTAATAGTAGAAGATG | pAH0141 | This study |
| MW2_arlR_3_D52E_R* | GGCAACATTAA**TTC**TAATATGATTAAATCATAGTAATG |  |  |
| MW2_arlR_3_D52E_F* | GATTTAATCATATTA**GAA**TTAATGTTGCCGTC |  |  |
| MW2_arlR_2_ScR | GAATTC***GAGCTC***GCAATTTACGTTTTGTCATCG |  |  |
| MW2_srrA_1_KF | CTTGAT***GGTACC*AGGAGGAAATT**ATGTCGAACGAAATACTTATCGTAGATG | pAH0142 | This study |
| MW2_srrA_3_D53E_R* | GCAACATTAA**TTC**TAGTAGTATGCAAGCATAATTATTC |  |  |
| MW2_srrA_3_D53E_F* | CATACTACTA**GAA**TTAATGTTGCCTGAAATG |  |  |
| MW2_srrA_2_ScR | GAATTC***GAGCTC***CACAGTTTAATTACGACACTATTTAGC |  |  |
| MW2_phoP_1_KF | CTTGAT***GGTACC*AGGAGGAAATT**ATGTCGCAAAAAGTGTTGGTAG | pAH0143 | This study |
| MW2_phoP_3_D53E_R* | GTAGCATAAC**TTC**TAAAATAATTAAATCTGGCTG |  |  |
| MW2_phoP_3_D53E_F* | ATTTAATTATTTTA**GAA**GTTATGCTACCTAAAAAAGATG |  |  |
| MW2_phoP_2_ScR | GAATTC***GAGCTC***GGTGAAACTTCATCATTGTTCTTTAGG |  |  |
| MW2_airR_1_KF | CTTGAT***GGTACC*AGGAGGAAATT**ATGAACAAAGTAATATTAGTAGATGACCATTATATTG | pAH0144 | This study |
| MW2_airR_3_D55E_R* | GGCATCACTAA**TTC**TAATAGCACAATATCAGGGTG |  |  |
| MW2_airR_3_D55E_F* | GATATTGTGCTATTA**GAA**TTAGTGATGCCTGGC |  |  |
| MW2_airR_2_ScR | GAATTC***GAGCTC***ATTCTAAATCAACTTATTTTCCATTG |  |  |
| MW2_vraR_1_KF | CTTGAT***GGTACC*AGGAGGAAATT**ATGACGATTAAAGTATTGTTTGTGGATG | pAH0145 | This study |
| MW2_vraR_3_D55E_R* | CATAAGTAA**TTC**CATTAAAATTAAATCTGGCTTCAAC |  |  |
| MW2_vraR_3_D55E_F* | ATTTAATTTTAATG**GAA**TTACTTATGGAAGACATGG |  |  |
| MW2_vraR_2_ScR | GAATTC***GAGCTC***AATACGAACTATTGAATTAAATTATGTTGG |  |  |
| MW2_agrA_1_KF | CTTGAT***GGTACC*AGGAGGAAATT**ATGAAAATTTTCATTTGCGAAGAC | pAH0146 | This study |
| MW2_agrA_3_D59E_R* | GTTGAAAGTTGAAT**TTC**TAAAAAGTAACAGC |  |  |
| MW2_agrA_3_D59E_F* | TTACTTTTTA**GAA**ATTCAACTTTCAACTGATATTAATG |  |  |
| MW2_agrA_2_ScR | GAATTC***GAGCTC***GAATACGCCGTTAACTGACTTTATTATC |  |  |
| MW2_kdpE_1_KF | CTTGAT***GGTACC*AGGAGGAAATT**ATGCAATCTAAAATATTGATAATTGAAGATG | pAH0147 | This study |
| MW2_kdpE_3_D53E_R* | GGTAAACCTAA**TTC**TAATAAAATGACATCTGG |  |  |
| MW2_kdpE_3_D53E_F* | GATGTCATTTTATTA**GAA**TTAGGTTTACCAGATAAAG |  |  |
| MW2_kdpE_2_ScR | GAATTC***GAGCTC***TGCCAATCATTCGTTTAATGG |  |  |
| MW2_hssR_1_KF | CTTGAT***GGTACC*AGGAGGAAATT**ATGGTGCAATGTCTTGTTGTCG | pAH0148 | This study |
| MW2_hssR_3_D52E_R* | CATCCATCATAAT**TTC**TACCACTGCAATATCG |  |  |
| MW2_hssR_3_D52E_F* | GCAGTGGTA**GAA**ATTATGATGGATGGTATGGAC |  |  |
| MW2_hssR_2_ScR | GAATTC***GAGCTC***GCAATTCTAGCATAGAGTGTTTTAAAC |  |  |
| MW2_nreC_1_KF | CTTGAT***GGTACC*AGGAGGAAATT**ATGAAAATAGTCATTGCCGATGATCAC | pAH0149 | This study |
| MW2_nreC_3_D53E_R* | CATACTTAA**TTC**CATTAGTAACACATCAGGTTTATATTC |  |  |
| MW2_nreC_3_D53E_F* | GTGTTACTAATG**GAA**TTAAGTATGCCACC |  |  |
| MW2_nreC_2_ScR | GAATTC***GAGCTC***TCGCGCTTTTATACTGAATTATATTAATTTC |  |  |
| MW2_braR_1_KF | CTTGAT***GGTACC*AGGAGGAAATT**ATGAAAATATTAATTGTTGAAGATGATTTTGTTATAG | pAH0150 | This study |
| MW2_braR_3_D51E_R* | CAAATTAAT**TTC**TAGCAATACAAGCTGAGGTTGAT |  |  |
| MW2_braR_3_D51E_F* | CAGCTTGTATTGCTA**GAA**ATTAATTTGCCAACG |  |  |
| MW2_braR_2_ScR | GAATTC***GAGCTC***GAAAGGTCATTGCATTCACCCTATAC |  |  |
| Sequencing primers | | | |
| pRMC2_US_F | CTCCTTTTTGTTGACATTATATCATTG |  | This study |
| pKX-DS-R | CGAAAGGGGGATGTGCTGC |  | (6) |
| Nre 3xflag epitope insertion |  | |  |
| PCad.infusion.2 | AGCTGGCGGCCGCT***gcatgC***GCACTTATTCAAGTGTATTT | pCN51::*nreCB*-3XFLAG | This study |
| Nre_infusion_rv1 | *tgtagtc*AAACTCTAataatttctttt |  |  |
| Nre_infusion_fw2 | AGAGTTT*gactacaaagaccatgacgg* |  |  |
| Nre_infusion_rv2 | TAATTCATCT***GGATCC***AATTTCAAACTCTA*tttatcgtcgtcatctttgtag* |  |  |
| Nre_pAH149_fw1 | gg***GGTACC*AGGAGGAAATT**atgaaaatag | pRMC2::*nreC*-3XFLAG | This study |
| Nre_pAH149_rv2 | gg***GAATTC****ctatttatcgtcgtcatctttgtag* |  |  |
|  | |  |  |
| Transcriptional expression of target genes promoters | | | |
| P_atl_MW937_fw | gg***GTCGAC***AATCTGAATCTATTCACCTCATTGG | pCN52::P*atl*::*gfp* | Mod. from (7) |
| P_atl_rv | gg***GGTACC***tctatttattactcctaacatttattaattattactaac |  | This study |
| P_UhpT_fw | gg***GTCGAC***agtacataaatatgtttctaagtatgtg | pCN52::P*uhpT*::*gfp* | Mod. from (8) |
| P_uhpT_rv | gg***GGTACC***ctctgtcacctcaatcattttc |  | This study |
| P_lrgA_fw | gg***GTCGAC***cgataaaattcacatgttaaagc | pCN52::P*lrgA*::*gfp* | Mod. from (9) |
| P_lrgA_rv | gg***GGTACC***cgtttgatttaactaaagtatagatgg |  |  |
| P_mprF_fw | gtc***GTCGAC***Gtatagataaccatattgttc | pCN52::P*mprF*::*gfp* | Mod. from (10) |
| P_mprF_rv | ggt***GGTACC***tgattcattttttcacatca |  |  |
| P_efb_fw | ***GTCGAC***aaggatttctaaagtcataaattatatc | pCN52::P*efb*::*gfp* | This study |
| P_efb_rv | ***GGTACC***aattaataacacctatcctcaaat |  |  |
| P_MW1206_fw | ctgcag***gtcgac***tttatttgtaaggagtctcgattatagagg | pCN52::P*MW1206*::*gfp* | This study |
| P_MW1206_rv | cctcct***ggatcc***tttaatcacctctattttttccttaatttaatattagtaaatttattag |  |  |
| P_mgrA_fw | ATGCCTGCAGGTCGACGTCCCCTTTTAAAGCAATGGC | pCN52::P*mgrA*::*gfp* | Mod. from (11) |
| P_mgrA_rv | ACGAATTCGAGCTCGAATAAGAATATCCATAATTAACGGATTTTTGGGTAGT |  |  |
| P_qox_fw | ***GTCGAC***gctattctcaactttcctttta | pCN52::P*qox*::*gfp* | This study |
| P_qox_rv | ***GGTACC***tttttgacctcctaatactaca |  |  |
| P_pstS_fw | gg***GTCGAC***TACATGTTAATACGTAGTATTAATGGCGAGAC | pCN52::P*pstS*::*gfp* | Mod. from (12) |
| P_pstS_rv | gg***GGTACC***tgaaatatcctccctgtatgaacaacaa |  |  |
| P_MW2540_fw | gg***GTCGAC***atttaaaattgagcgcttagtatgc | pCN52::*PMW2540*::*gfp* | This study |
| P_MW2540_rv | gg***GGTACC***atgttttcacctcaataaacttgaaatag |  |  |
| P_MW1056_fw | gg***GTCGAC***ggcttagaaggccattgctc | pCN52::*PMW1056*::*gfp* | Mod. from (13) |
| P_MW1056_rv | gg***GGTACC***cggtatctttaattgcgttaaataaac |  |  |
| P_cap8A_fw | gg***GTCGAC***gcatttgaagatcaatgtacaac | pCN52::P*cap8*::*gfp* | Mod. from (14) |
| P_cap8A_rv | gg***GGTACC***GTATTTACAAGTTGAATATTACTTTG |  |  |
| P_hrtA_fw | gg***GTCGAC***GCACCATAGCTATAAACTCC | pCN52::P*hrtA*::*gfp* | Mod. from (15) |
| P_hrtA_rv | gg***GGTACC***TTCATATCGATTCACTTCTCC |  |  |
| P_narG_nasF_fw | gg***GTCGAC***caacttctaatccgactcagc | pCN52::P*narG*::*gfp* | Mod. from (16) |
| P_narG_nasF_rv | gg***GGTACC***atttatatcctcctacgtataaaaatacgatg |  |  |
| P_ctsR_fw | gg***GTCGAC***aagaatggtggttgcttaggatcg | pCN52::P*ctsR*::*gfp* | Mod. from (17) |
| P_ctsR_rv | gg***GGTACC***tatatcacccctttttgaccttc |  |  |

Restriction sites are highlighted in bold and italics, ribosomal binding sites inserted upstream of the relevant phosphomimetic TCS RR are highlighted in bold and underlined, phosphomimetic codon substitutions are shades.

**References**

1. **Baba T**, **Takeuchi F**, **Kuroda M**, **Yuzawa H**, **Aoki KI**, **Oguchi A**, **Nagai Y**, **Iwama N**, **Asano K**, **Naimi T**, **Kuroda H**, **Cui L**, **Yamamoto K**, **Hiramatsu K**. 2002. Genome and virulence determinants of high virulence community-acquired MRSA. The Lancet **359**:1819–1827.

2. **Villanueva M**, **García B**, **Valle J**, **Rapun B**, **Ruiz de Los Mozos I**, **Solano C**, **Marti M**, **Penadés JR**, **Toledo-Arana A**, **Lasa I**. 2018. Sensory deprivation in *Staphylococcus aureus*. Nat Commun **9**:523.

3. **Burgui S**, **Gil C**, **Solano C**, **Lasa I**, **Valle J**. 2018. A Systematic Evaluation of the Two-Component Systems Network Reveals That ArlRS Is a Key Regulator of Catheter Colonization by *Staphylococcus aureus*. Front Microbiol **9**:6069–11.

4. **Charpentier E**, **Anton AI**, **Barry P**, **Alfonso B**, **Fang Y**, **Novick RP**. 2004. Novel cassette-based shuttle vector system for Gram-positive bacteria. Appl Environ Microbiol **70**:6076–6085.

5. **Corrigan RM**, **Foster TJ**. 2009. An improved tetracycline-inducible expression vector for *Staphylococcus aureus*. Plasmid **61**:126–129.

6. **Haag AF**, **Wehmeier S**, **Muszyński A**, **Kerscher B**, **Fletcher V**, **Berry SH**, **Hold GL**, **Carlson RW**, **Ferguson GP**. 2011. Biochemical characterization of *Sinorhizobium meliloti* mutants reveals gene products involved in the biosynthesis of the unusual lipid A very long-chain fatty acid. J Biol Chem **286**:17455–17466.

7. **Monk IR**, **Shaikh N**, **Begg SL**, **Gajdiss M**, **Sharkey LKR**, **Lee JYH**, **Pidot SJ**, **Seemann T**, **Kuiper M**, **Winnen B**, **Hvorup R**, **Collins BM**, **Bierbaum G**, **Udagedara SR**, **Morey JR**, **Pulyani N**, **Howden BP**, **Maher MJ**, **McDevitt CA**, **King GF**, **Stinear TP**. 2019. Zinc-binding to the cytoplasmic PAS domain regulates the essential WalK histidine kinase of *Staphylococcus aureus*. Nat Commun **10**:3067.

8. **Yang Y**, **Sun H**, **Liu X**, **Wang M**, **Xue T**, **Sun B**. 2015. Regulatory mechanism of the three-component system HptRSA in glucose-6-phosphate uptake in *Staphylococcus aureus*. Medical Microbiology and Immunology **205**:241–253.

9. **Moormeier DE**, **Endres JL**, **Mann EE**, **Sadykov MR**, **Horswill AR**, **Rice KC**, **Fey PD**, **Bayles KW**. 2013. Use of microfluidic technology to analyze gene expression during *Staphylococcus aureus* biofilm formation reveals distinct physiological niches. Appl Environ Microbiol **79**:3413–3424.

10. **Falord M**, **Mäder U**, **Hiron A**, **Dbarbouillé M**, **Msadek T**. 2011. Investigation of the *Staphylococcus aureus* GraSR regulon reveals novel links to virulence, stress response and cell wall signal transduction pathways. PLoS ONE **6**:e21323.

11. **Crosby HA**, **Schlievert PM**, **Merriman JA**, **King JM**, **Salgado-Pabón W**, **Horswill AR**. 2016. The *Staphylococcus aureus* Global Regulator MgrA Modulates Clumping and Virulence by Controlling Surface Protein Expression. PLoS Pathog **12**:e1005604.

12. **Kelliher JL**, **Radin JN**, **Kehl-Fie TE**. 2018. PhoPR contributes to *Staphylococcus aureus* growth during phosphate starvation and pathogenesis in an environment- specific manner. Infect Immun **86**.

13. **Periasamy S**, **Joo H-S**, **Duong AC**, **Bach THL**, **Tan VY**, **Chatterjee SS**, **Cheung GYC**, **Otto M**. 2012. How *Staphylococcus aureus* biofilms develop their characteristic structure. Proc Natl Acad Sci USA **109**:1281–1286.

14. **Cocchiaro JL**, **Gómez MI**, **Risley A**, **Solinga R**, **Sordelli DO**, **Lee JC**. 2006. Molecular characterization of the capsule locus from non-typeable *Staphylococcus aureus*. Mol Microbiol **59**:948–960.

15. **Stauff DL**, **Torres VJ**, **Skaar EP**. 2007. Signaling and DNA-binding activities of the *Staphylococcus aureus* HssR-HssS two-component system required for heme sensing. J Biol Chem **282**:26111–26121.

16. **Yan M**, **Yu C**, **Yang J**, **Ji Y**. 2011. The essential two-component system YhcSR is involved in regulation of the nitrate respiratory pathway of *Staphylococcus aureus.* J Bacteriol **193**:1799–1805.

17. **Wozniak DJ**, **Tiwari KB**, **Soufan R**, **Jayaswal RK**. 2012. The mcsB gene of the clpC operon is required for stress tolerance and virulence in *Staphylococcus aureus*. Microbiology **158**:2568–2576.
